# Impact of developmental temperature on neural growth, connectivity, and function

**DOI:** 10.1126/sciadv.adp9587

**Published:** 2025-01-15

**Authors:** Pascal Züfle, Leticia L. Batista, Sofia C. Brandão, Giovanni D’Uva, Christian Daniel, Carlotta Martelli

**Affiliations:** ^1^ Johannes Gutenberg University, Mainz, Germany.; ^2^ Institute for Quantitative and Computational Biosciences, Mainz, Germany.

## Abstract

Environmental temperature dictates the developmental pace of poikilothermic animals. In *Drosophila*, slower development at lower temperatures results in higher brain connectivity, but the generality of such scaling across temperatures and brain regions and its impact on function are unclear. Here, we show that brain connectivity scales continuously across temperatures, in agreement with a first-principle model that postulates different metabolic constraints for the growth of the brain and the organism. The model predicts brain wiring under temperature cycles and the nonuniform temporal scaling of neural development across temperatures. Developmental temperature has notable effects on odor-driven behavior. Dissecting the circuit architecture and function of neurons in the olfactory pathway, we demonstrate that developmental temperature does not alter odor encoding in first- and second-order neurons, but it shifts the specificity of connections onto third-order neurons that mediate innate behaviors. We conclude that while some circuit computations are robust to the effects of developmental temperature on wiring, others exhibit phenotypic plasticity with possible adaptive advantages.

## Teaser

Developmental temperature tunes brain wiring with consequences for circuit function and animal behavior.

## INTRODUCTION

The wiring of the nervous system follows a complex genetic plan during development. However, because of stochastic processes and environmental factors, genetically identical individuals seldomly show the same phenotypic outcome (*1*). Temperature is the environmental factor with the broadest effects in biology, as it determines the rates of all biophysical reactions of an organism (*2*). In poikilothermic animals, such as insects, worms, fish, amphibians, and reptiles, temperature determines the speed of development. Mathematical theories of growth have shown that developmental times scale exponentially with temperature because of a constraint imposed by the rate-limiting metabolic reaction (*3*). The development of the nervous system is certainly not exempted by the effects of temperature.

It has been widely reported that development at different temperatures correlates with variation in behavioral phenotypes with examples in amphibians (*4*), reptiles (*5*–*7*), bees (*8*, *9*), ants (*10*), and fruit flies (*11*, *12*). Social insects invest a lot of resources to keep their broods at the correct temperature throughout daily and seasonal cycles. Even small variations in developmental temperature can affect learning in bees (*13*) and the synaptic organization of key brain areas for learning (*12*, *14*, *15*). Therefore, temperature does not just determine the speed, but also the outcome of development, although the mechanistic bases of this phenotypic variation are largely unknown.

A recent study in *Drosophila* reported that the number of synaptic connections between neurons of the visual system inversely correlate to temperature, i.e., when flies develop at lower temperature, these neurons make more synapses and have more postsynaptic partners (*16*). This leads to the questions of whether synaptic scaling occurs similarly throughout the brain and what consequences it has on neural computations and behavior. Answering these questions is key not only to predict the consequences of temperature changes on animal behavior in the wild, but also to fully understand the organization of development in poikilothermic animals.

Here, we investigate how developmental temperature affects the wiring and function of the fly olfactory circuit. We show that development at lower temperatures leads to higher connectivity at all stages of olfactory processing and changes the neural composition of local circuits. To explain these findings, we develop a first-principle theory that postulates the existence of different metabolic constraints for the growth of the whole animal and the brain. This theoretical framework explains changes in connectivity associated with different developmental conditions and predicts the nonuniform temporal scaling of neural growth across temperatures. Temperature manipulation during pupal development affects odor approach behavior, even when flies are adapted for 10 days at a common temperature. By measuring odor responses in first- and second-order olfactory neurons, we conclude that the behavioral differences cannot be attributed to a change in odor representations or sensitivity, which are robust to developmental temperature. Instead, we provide evidence that these odor representations are processed differently in downstream circuits that drive innate behaviors. Our results therefore indicate that the effect of developmental temperature on neural circuit function is not due to a general scaling in connectivity, but to a change in connection specificity across brain regions.

## RESULTS

### Impact of developmental temperature on the connectivity of an olfactory glomerulus

**Fig. 1.**
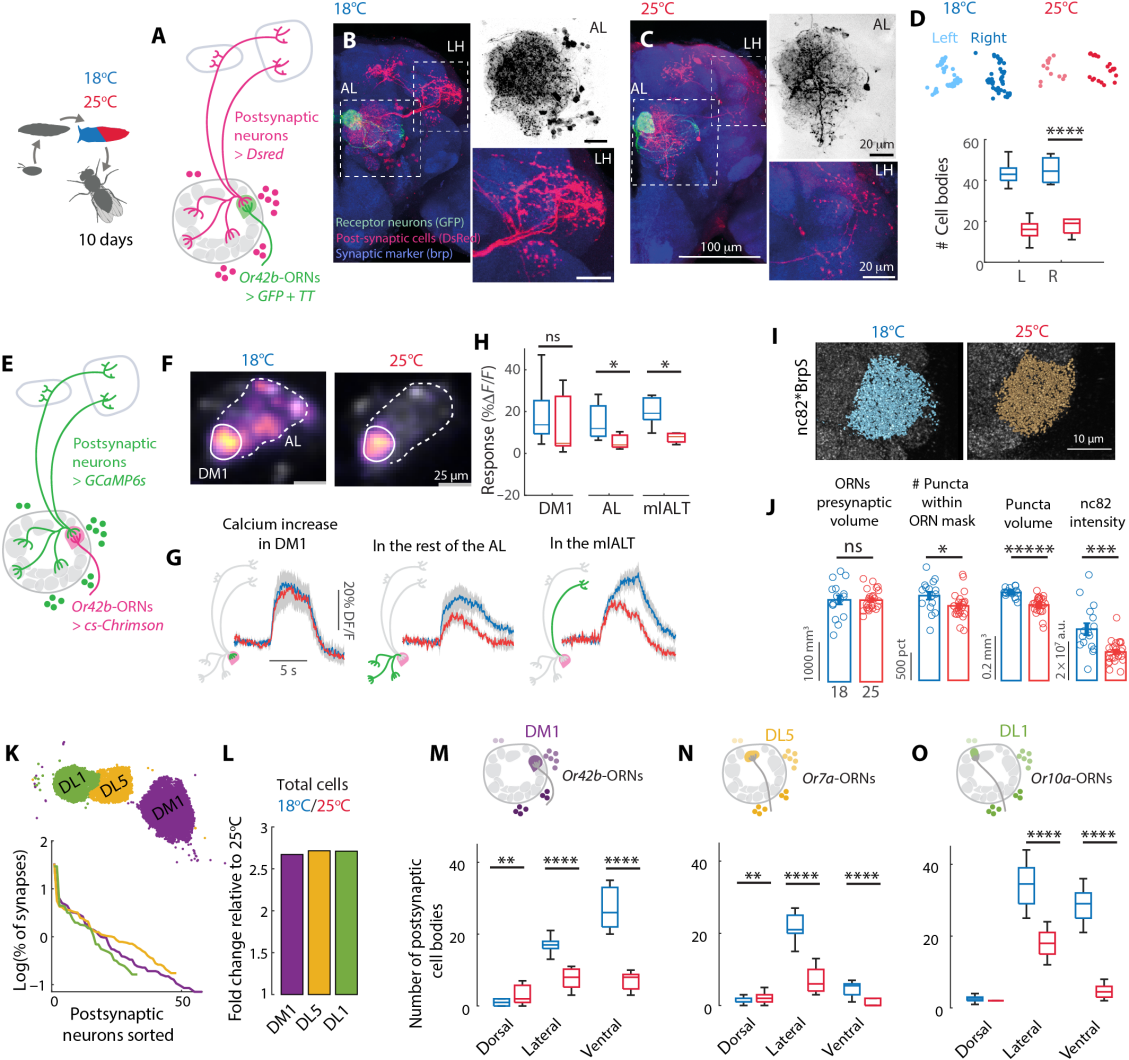
Developmental temperature affects the connectivity of ORNs in the AL. (**A**) Schematics of the *trans*-Tango tool. (**B** and **C**) Example brains from flies developed at 18° and 25°C. Immunostaining labels ORNs that express green fluorescent protein (GFP, green), postsynaptic partners expressing dsRed (red), and all synapses (Brp, blue). (**D**) Cell body locations of postsynaptic partners in left (L) and right (R) hemispheres in two individual flies and corresponding boxplot, Kruskal-Wallis, *P* < 10^−7^, *n* = 10 (18°C) and 10 (25°C). (**E**) Schematics of the experiment: The usual *trans*-Tango reporter dsRed was replaced by GCaMP6s, and activity of presynaptic neurons was induced by CsChrimson. (**F**) Example responses for flies developed at different temperatures. Color bar indicates Δ*F*/*F* calculated for each pixel. White lines indicate the region of interests used for quantification. (**G**) Optogenetic response to 0.05 mW/mm^2^ quantified in the DM1 glomerulus, in the rest of the AL and in the mlALT. (**H**) Boxplot quantifies the response in 9 s following stimulus onset. Significant differences were observed in the AL [Kruskal-Wallis, *P* = 0.02, *n* = 4 (18°C), and 7 (25°C)] and in the mlALT [*P* = 0.01, *n* = 4 (18°C), and n = 7 (25°C)]. (**I**) Anti-Brp staining of synaptic puncta and 3D reconstruction within the ORN mask created from Brp^Short^-GFP fluorescence. (**J**) Volume of the Brp^Short^-mask, number of puncta, puncta volume, and intensity within the ORN mask (error bars indicate SEM, *n* = 16 hemibrains at 18°C and *n* = 25 at 25°C, Kruskal-Wallis test). (**K**) Allocation of ORN output synapses onto individual partners for the three glomeruli, as quantified from electron microscopy (EM) data (see Materials and Methods). Top: Spatial location of the synapses analyzed. (**L**) Fold change in number of synaptic partners relative to 25°C. (**M** to **O**) Number of synaptic partners labeled by trans-Tango in different cell clusters of the AL. Sample size at 18° and 25°C: DM1 *n* = 20 and 23, DL5 *n* = 10 and 10, DL1 *n* = 10 and 10, Kruskal-Wallis. *P < 0.05, **P < 10^−2^, ***P < 10^−3^, *****P < 10^−5^. Boxplots indicate median and quartiles, and whiskers indicate maximum and minimum values.

To investigate the effects of temperature on the development of similar, but functionally distinct neural circuits, we focus on the olfactory system of *Drosophila*. Olfaction mediates key behaviors in animals, including foraging and mating, plays a major role in the organization of animal societies, and supports adaptation to the environment. We set out to investigate the effect of developmental temperature on the wiring of olfactory receptor neurons (ORNs) within the antennal lobe (AL), the main olfactory area in the insect brain. We used trans-Tango (*17*), a genetic tool for trans-synaptic labeling, to analyze postsynaptic neurons of *Or42b*-ORNs, i.e., ORNs that express the odorant receptor *Or42b* and target the glomerulus DM1 (Fig. 1A). Flies were developed at either 18° or 25°C between the larval stage L3 and the end of pupal development (which we name P-100%, because the absolute developmental time depends on temperature). Flies were then kept at 25°C for 10 days before dissection, to minimize acute effects of temperature on brain wiring and transgene expression. Notably more postsynaptic neurons were labeled in flies that developed at 18°C, as suggested by denser innervations in both the AL and downstream areas (Fig. 1, B and C). We quantified these differences by counting the cell bodies of the postsynaptic cells in each fly, which were more than double at 18°C as compared to 25°C (Fig. 1D).

To demonstrate that the anatomically labeled postsynaptic neurons are functionally connected to the ORNs, we modified the *trans*-Tango experiment to express a calcium reporter (GCaMP6s) in the postsynaptic neurons, and CsChrimson in *Or42b*-ORNs (Fig. 1E). This allowed us to optogenetically activate *Or42b*-ORNs and measure the response of their postsynaptic partners. Optogenetic activation of ORNs is sufficient to induce postsynaptic responses in a dose-dependent manner (fig. S1A). We then measured calcium signals in the *trans*-Tango experiment upon optogenetic activation of the ORNs. Calcium transients measured within the DM1 glomerulus mostly report activity from the single uniglomerular projection neuron (uPN, which receives most of the ORN synapses) and do not differ between the two temperatures (Fig. 1G and fig. S1A). However, activation in the rest of the AL is higher in flies developed at 18°C (Fig. 1, F to H), which is consistent with a larger number of multiglomerular neurons being connected to DM1. Moreover, activity is stronger in the mediolateral AL track (mlALT; Fig. 1, G and H) through which a class of multiglomerular inhibitory PNs (vPNs) project their axons to the lateral horn (LH). Therefore, the *trans*-Tango labeled neurons are functionally connected to *Or42b*-ORNs; moreover, ORNs drive more activity in flies developed at 18°C, which is consistent with the recruitment of new synaptic partners compared to higher temperatures.

The observed phenotypes are unlikely caused by the effect of temperature on the expression strength of the UAS-GAL4 system, as higher temperature should increase (and not decrease) the activity of this yeast-derived expression system (*18*). This was also confirmed by a quantification of the expression of the *trans*-Tango components [figure S3 in (*17*)]. Although we cannot exclude that other aspects of this tool might be temperature dependent, these effects should be minimized by our protocol because we have kept adult flies at a reference temperature of 25°C for 10 days before dissection. Furthermore, we show that the differences between temperatures are consistent across ages (fig. S1B) and that *trans*-Tango can potentially even report a decrease in connectivity induced by antennal clipping (fig. S1C), together arguing that this is a suitable tool to track changes in connectivity related to developmental temperature.

Higher connection at lower temperatures could result from either a different distribution of synapses across postsynaptic partners, an increase in synapse number, or both. To quantify the number of synapses, we expressed a green fluorescent protein (GFP)–tagged Brp^Short^ in *Or42b*-ORNs to label presynapses specifically in these cells and stained the endogenous Brp to count synaptic puncta (*19*) (nc82; Fig. 1I). The *Or42b*-ORNs presynaptic volume defined by the Brp^Short^ signal was consistent across the two developmental temperatures, indicating no change in the glomerulus volume (Fig. 1J). However, flies developed at 18°C had higher Brp^Short^ intensity, an increased number of nc82-labeled Brp puncta within the presynaptic volume, larger puncta volumes, and stronger intensity (Fig. 1J). A similar increase was observed when considering the whole volume of the DM1 glomerulus, which includes connections between other neuron types (fig. S1D). Together, these results demonstrate that a lower developmental temperature leads both to more synapses, larger active zones, and more synaptic partners which are functionally connected to the ORNs.

### Temperature-induced wiring differences across olfactory glomeruli

We asked whether developmental temperature scales connectivity similarly across glomeruli. *Trans*-Tango likely reports synaptic partners with synapse counts above a certain threshold. We used the hemibrain connectome data (*20*, *21*) from flies developed at 25°C to quantify the number of synapses per synaptic partner in *Or42b*-ORNs (fig. S1E). Comparing this with the number of postsynaptic neurons reported by *trans*-Tango at 25°C, we estimated that *trans*-Tango reports connected neurons with more than 10 synapses (total across ORNs of the same type; fig. S1E). Development at 18°C likely increases connectivity with available partners, which perhaps are already connected with few synapses at 25°C (fig. S1E). To test this hypothesis, we considered two additional ORN types (expressing *Or7a* and *Or10a*) that, based on connectome data, distribute similarly their synapses onto synaptic partners (Fig. 1K). We reasoned that the same fold change in synaptic partners should be expected for these ORN types if lower temperature shifted the overall connectivity up. Consistently, the fold change in postsynaptic partners between 18° and 25°C was remarkably similar for the three glomeruli (Fig. 1L).

We next looked at the identity of the postsynaptic partners of these ORNs. We hypothesized that, despite a similar scaling of connectivity, the identity of synaptic partners recruited by these different ORNs should depend on available neurons in the relevant glomerulus volume. AL neurons postsynaptic to ORNs have their cell bodies distributed in four clusters (*22*). We used cell body location as a proxy for cell type. On the basis of *trans*-Tango experiments, there were more neurons postsynaptic to *Or42b*-ORNs in the ventral and ventrolateral clusters (Fig. 1M) that host cell bodies of 66.6% of all multiglomerular PNs (mPNs) (*23*) including vPNs (*24*–*27*). This suggests that *Or42b*-ORNs might drive more activity in this population of neurons when flies develop at 18°C as compared to 25°C. This anatomical finding is consistent with increased calcium activity in the mlALT, which is used by these neurons to reach the LH (Fig. 1H). On the contrary, in glomeruli DL5 and DL1, ORNs increase connectivity more prominently with neurons of the lateral cluster (Fig. 1, N and O) that contains a large population of LNs, and mPNs mostly of unknown function (*28*). An analysis of the connectome data confirms that more mPNs innervate the DM1 glomerulus than the DL1 or DL5 glomeruli, where we instead find a larger percentage of LNs, which are mostly inhibitory (S1F). These findings suggest different functional consequences of developmental temperature in different glomeruli. In general, we predict a stronger recruitment of local inhibition in all glomeruli at lower temperatures. For the DM1 glomerulus, we predict it might drive activity in more mPNs that send axons to the LH with potential consequences for behavior.

### Scaling of brain connectivity across temperatures

**Fig. 2.**
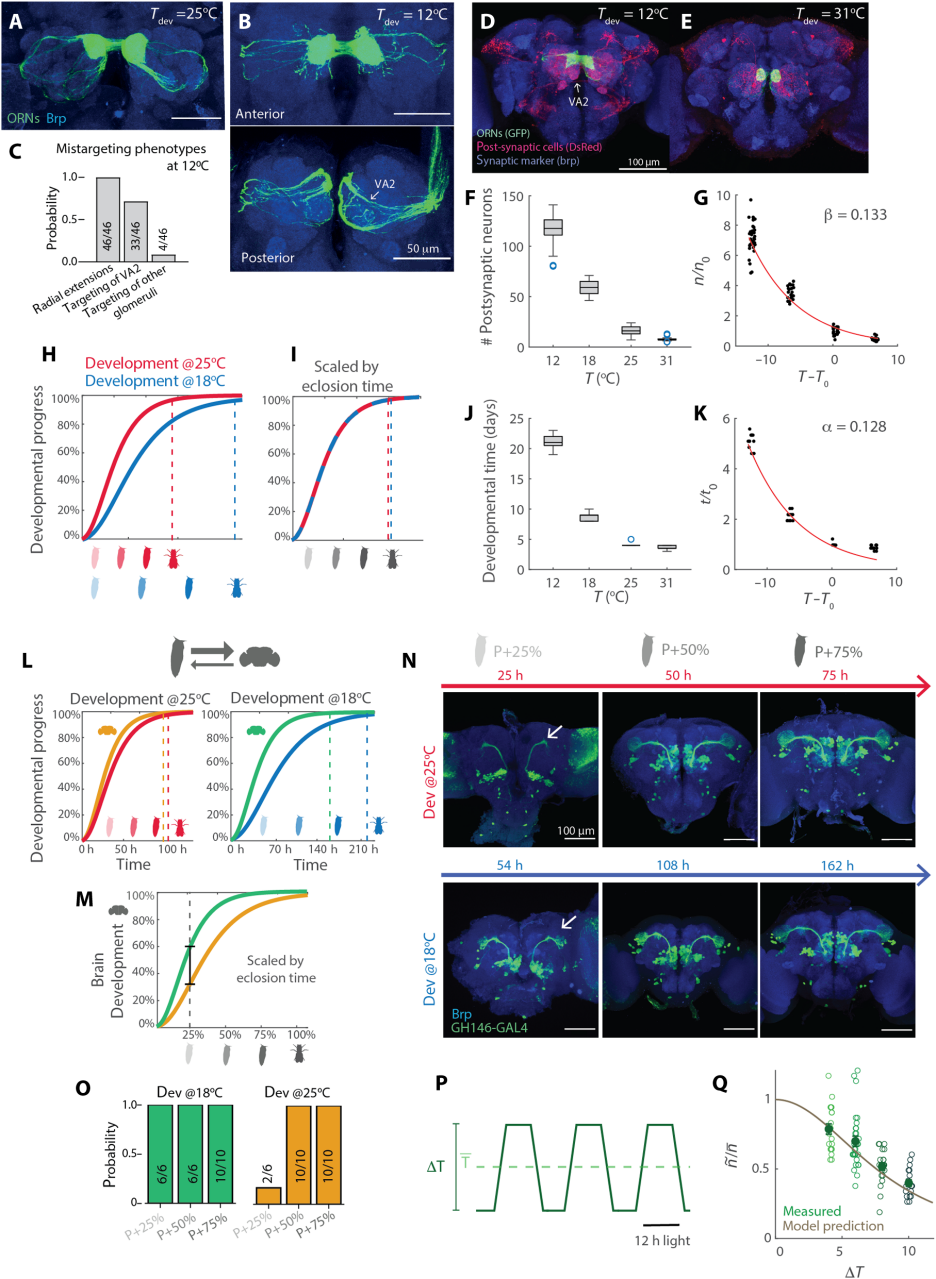
A first-principle model for network connectivity at different temperatures. (**A** and **B**) *Or42b*-ORN axons labeled by GFP in flies developed at 25° and 12°C. (**C**) Probability of the mistargeting phenotypes at 12°C. Sample size is indicated on the bars. (**D** and **E**) Example brains of flies expressing *trans*-Tango under control of *Or42b-Gal4* developed at 12° or 31°C. (**F**) Boxplot of the number of postsynaptic partners as a function of developmental temperature. (**G**) Single data points as in (F), normalized by the mean number of neurons connected at a reference temperature (25°C) and plotted as a function of the difference from the reference temperature (*n* = 20 to 38 hemibrains). The line indicates the exponential fit. (**H**) Hypothetical growth curve showing the effects of developmental temperature. (**I**) Same as (H) but normalized by eclosion time. (**J**) Developmental times for the four temperature regimes. (**K**) Fold change in developmental time with respect to the temperature difference from 25°C and exponential fit (*n* = 8 to 20). (**L**) Schematics of the growth model showing percentage of development as a function of time for the brain and whole animal at two developmental temperatures. Parameters of the growth dynamics are hypothetical. (**M**) Brain growth curved scaled by body developmental time. (**N**) Staining of PNs labeled by *GH146* at three developmental times corresponding to 25, 50, and 75% of ontogenesis at the two temperatures. The arrow indicates the LH. (**O**) Probability that PNs axon terminals innervate the LH (*n* = 6 to 10 hemibrains). (**P**) Schematics of the temperature and light cycles. (**Q**) Fold change with respect to the mean temperature in the number of synaptic partners for development on temperature cycles of amplitude Δ*T* (*n* = 18 to 30 hemibrains). The solid line indicates model prediction (see Materials and Methods).

Next, we asked whether synaptic connectivity scales continuously across a wider range of temperatures. *Drosophila melanogaster* lives in different climates, with a thermal range between 11° and 31°C (*29*). The extreme conditions are highly stressful, and if persistent, female reproductive success would approach zero (*30*). However, flies were viable when temperature shifts were restricted to pupal development. We first looked at the anatomy of the ORNs axons targeting the DM1 glomerulus in flies developed at 12°C. Compared to 25°C, ORNs grow axonal extensions protruding radially from the glomerulus (Fig. 2, A and B) and innervate a more anterior glomerulus, VA2, that is normally not targeted by this receptor type (Fig. 2, B and C). We used *trans*-Tango to characterize connectivity patterns of *Or42b*-ORNs and its postsynaptic partners in flies that developed at extreme temperatures (12 to 31°C). In flies developed at 12°C, ORNs established a larger number of synaptic partners, including neurons that target regions outside the canonical olfactory pathway (Fig. 2, D and F), including neurons that arborize in the saddle (details and controls in fig. S2A). Development at lower temperatures therefore leads to the recruitment of new synaptic partners. Flies developed at 31°C instead showed very few neurons connected to *Or42b*-ORNs (Fig. 2, E and F). Across all developmental temperatures tested, the number of ORNs was constant (fig. S2B), but the number of synaptic partners scaled exponentially with temperature (Fig. 2G).

### A metabolic theory for brain wiring at different temperatures

To understand this scaling in connectivity, we took a theoretical approach. We started from the observation that temperature determines developmental time, which we define as the time of eclosion of the adult. We assumed that development follows a growth process whose rate depends on temperature, such that the adult ecloses later at lower temperatures (Fig. 2H). In general, one could expect that normalizing time by the adult eclosion should collapse the developmental process at the two temperatures on the same growth curve (Fig. 2I). This would be consistent with the model introduced by Gillooly *et al.* (*3*), which links growth rate to metabolism and temperature. The model postulates that the rate of development scales with temperature proportionally to the Boltzmann factor $e^{-\frac{E}{KT_{k}}}$ of a hypothetical rate-limiting metabolic reaction (*3*) (E being its activation energy). Assuming that flies eclose at a similar mass across temperatures, developmental time t should scale with the temperature T as $\frac{t}{t_{0}}=e^{-\frac{E}{KT_{a}^{2}}\left(T-T_{0}\right)}$ (see Materials and Methods), where $t_{0}$ is the developmental time at a reference temperature $T_{0}$ (here $T_{0}=25{}^{\circ}C$), E is the activation energy of the rate-limiting metabolic reaction, K is the Boltzmann constant, and $T_{a}$ is the water freezing point (273 K; see Materials and Methods). This model fits our experimental data, with a scaling factor for the developmental time ($\alpha =\frac{E}{KT_{a}^{2}}$) equal to 0.128(±0.009) (Fig. 2, J and K), which is in agreement with estimates in other animals (*3*). This simple exponential model fails to fit developmental times at the highest temperatures above 30°C (*31*); nonetheless, we took it as a starting point for our theoretical framework.

We reasoned that if the development of the neural circuit was limited by the same reaction rate as ontogenesis (development of the whole organism), then development should result in the same brain connectivity at all temperatures (Fig. 2I). If, otherwise, the development of the neural system was limited by a reaction with lower activation energy E′<E, then it should scale differently compared to body development when temperature changes (Fig. 2L). The neural circuit will therefore develop (extend axons and form synapses) at its own, faster pace, but for an amount of time that is determined by ontogenesis, therefore leading to a larger number of synapses. To calculate the fold change in connectivity, we assumed that axonal growth and synaptogenesis scale with a ^3^/_4_ power law: $\frac{\mathrm{dn}}{\mathrm{dt}}∼an^{\frac{3}{4}}$ (n indicating a measure of neuronal mass and synapses; see Materials and Methods), as for body mass (*3*). Many biological processes, including growth, scale allometrically with the ^3^/_4_ law (*32*, *33*). This phenomenological observation has received a first-principle explanation based on the fractal nature of the distribution of nutrients and resources in a three-dimensional (3D) volume by a space-filling network of branching tubes (*34*, *35*). Given the tree-like structure of axons, along which mitochondria and proteins need to be transported, and of other energy suppliers in the brain (such as trachea and glia), the ^3^/_4_ law is a reasonable assumption for the growth of the neural system. From this, we derived an analytical function for the scaling of synaptic connectivity with temperature: $\frac{n}{n_{0}}=e^{-4\frac{∆E}{KT_{a}^{2}}\left(T-T_{0}\right)}$ with ∆E=E−E′ (see Materials and Methods). We define $\beta =\frac{4∆E}{KT_{a}^{2}}$ as the scaling factor for synaptic connectivity. When ∆E=0, the same number of connections is established across temperatures. This law fits well the exponential change in connectivity in the olfactory circuit (Fig. 2K) with β=0.133(±0.011). From β and, we calculate the activation energy E′=0.61 eV of the rate-limiting metabolic reaction for brain development, which falls in the range of feasible value for the metabolism (*36*). It should be noted that β can, in principle, be different across subcircuits if their development and growth are differently regulated. In the visual system, Kiral *et al.* (*16*) reported a fold change of ~1.5 that corresponds to a smaller value of β compared to what we find in the olfactory system. Whether these differences are technical or biological remains to be determined, but the proposed model could easily accommodate differences across circuits.

### Nonuniform scaling of neural growth during development

Our model makes some predictions, which might be counterintuitive. First of all, by normalizing the time axis of the brain growth curves based on eclosion time (ontogenesis; Fig. 2L), the model predicts that the brain is in a more advanced developmental stage at lower as compared to higher temperatures (Fig. 2M). This implies that at proportional times of pupal metamorphosis (P-25%, P-50%, and P-75%), the branching anatomy of a given neuron type is expected to be more advanced in flies developed at 18°C, as compared to 25°C. We experimentally tested this prediction by looking at the advancement of PN development across times and temperatures. Consistent with the model prediction, we found that PN axons already targeted the LH at P-25% when flies developed at 18°C, while these axonal innervations were not yet visible at P-25% in flies developed at 25°C (Fig. 2, N and O), consistent with previous data (*37*). This prediction of the model is independent of the analysis of synaptic partners. Overall, the model and the experimental data agree that developmental temperature induces a nonuniform temporal scaling of neural circuit development as compared to ontogenesis.

### Brain wiring under temperature cycles

A second prediction of the model concerns development on diurnal temperature cycles. In more ecologically relevant conditions, an animal does not experience constant temperatures during development. Does development under cycling temperatures result in the same wiring outcome as for flies developed at the mean temperature? We first answered this question using our metabolic model. Integrating the growth equation over temperature cycles (see Materials and Methods), we derived that circuit connectivity scales inversely with the amplitude ∆T of the temperature fluctuations as: $\frac{\tilde{n}}{\bar{n}}={\left[\frac{\text{cosh}\left(\gamma ∆T\right)}{\text{cosh}\left(\alpha ∆T\right)}\right]}^{4}$, where $\tilde{n}$ and $\bar{n}$ are the number of connections for development on temperature cycles and at the mean temperature, and $\gamma =\alpha -\frac{\beta }{4}$ with α and β estimated from the fixed temperature experiments (Fig. 2, G and K). Because γ<α, fluctuating temperatures should always lead to less connections than at the mean temperature $\bar{T}$. An intuitive explanation of this result is to be found in the fact that the time spent at low and at high temperature does not lead to equal amount of development. Rather, the brain grows more in the high-temperature phase of the cycle compared to the low temperature phase. Therefore, the outcome is skewed toward the high-temperature phenotype, leading to less connections during cycles compared to the mean temperature. To test this experimentally, we developed flies on ecologically realistic temperature cycles with the same mean temperature but different amplitudes (20° to 28°, 18° to 30°, 16° to 32°, and 14 to 34°C; Fig. 2P). *Trans*-Tango experiments showed that these conditions lead to different connectivity of *Or42b*-ORNs in the AL, which make more synaptic partners when temperature cycles had smaller amplitudes, following the prediction of the theory (Fig. 2Q). Moreover, the temperature experienced after eclosion does not strongly affect connectivity (fig. S2C). We noticed that developmental times, however, seem to deviate from the theory prediction (fig. S2D), probably because the temperature fluctuations reach extreme values up to 34°C (Fig. 2K). Overall, we conclude that temperature-dependent wiring is consistent with a growth model with different rate-limiting metabolic reactions for the development of the whole organisms and for the development of neural circuits, although deviations to this rule might occur at extreme high temperatures.

### Impact of developmental temperature on odor-driven behavior

**Fig. 3.**
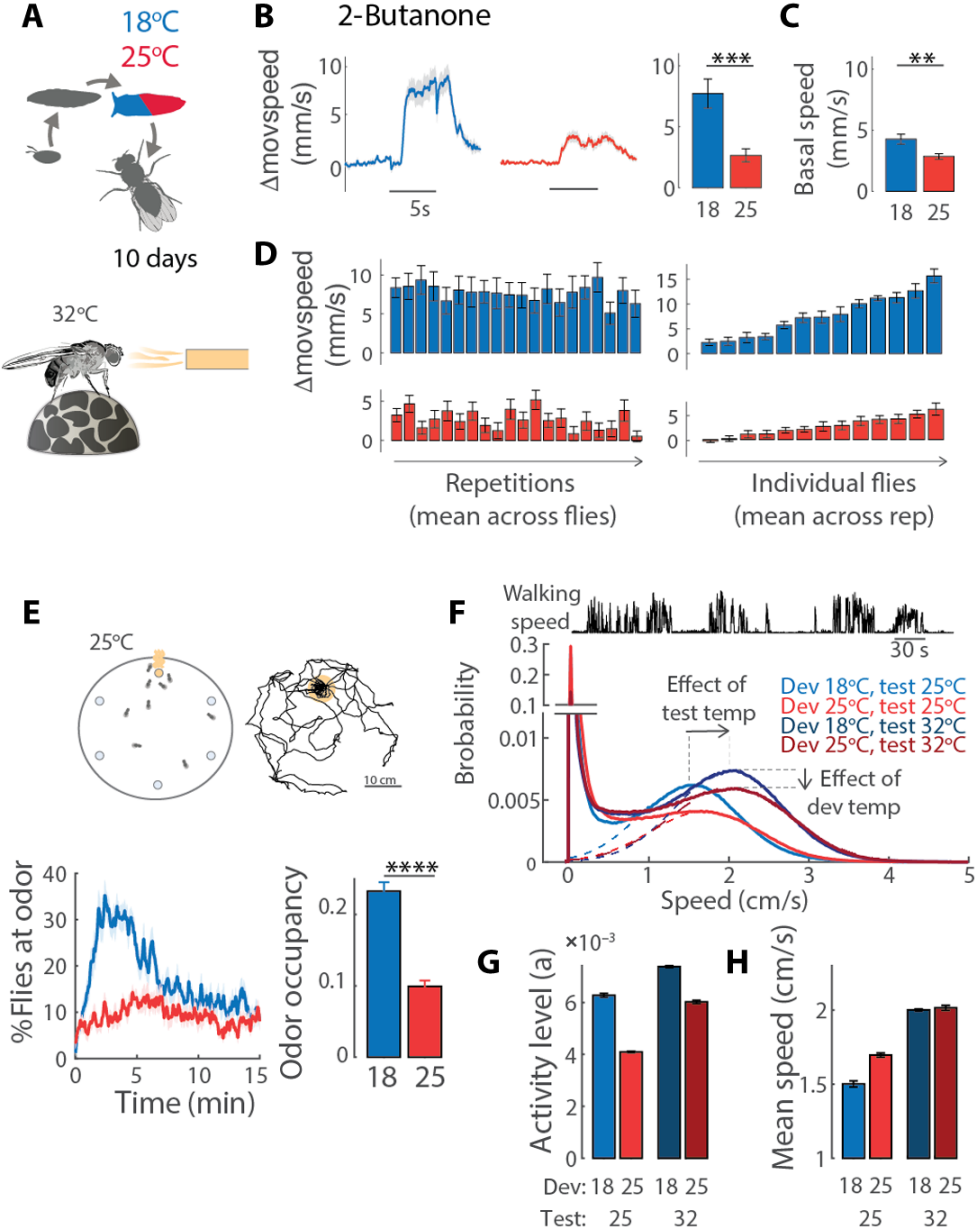
Developmental temperature affects odor-driven behavior. (**A**) Flies were kept at 25°C for 10 days before behavioral experiments. Odor response was tested at 32°C on a spherical treadmill (see Materials and Methods). (**B**) Odor response was calculated as change in moving speed as a function of time in response to 5-s stimulation (Δmovspeed = speed − basal speed); bar plot, mean change in moving speed within the first 2 s of stimulation [*P* < 10^−3^, *n* = 13 (18°C) and 13 (25°C)]. (**C**) Basal walking speed is estimated within 3 s before stimulus onset (*P* = 0.02). (**D**) Mean response to each consecutive stimuli repetitions averaged across all flies (right), and mean response of each individual fly across repetitions (left). (**E**) Top: Same protocol as in (A), but odor preference was tested at 25°C in a free walking assay consisting in a circular arena of 40-cm radius with an odor source randomly placed in one of six possible locations. Flies were tracked for 15 min (see Materials and Methods). Bottom: Average percentage of flies at the odor source and odor occupancy (bar plot). Shaded area and error bars indicate SEM [*P* < 10^−6^, *n* = 19 (18°C) and 20 (25°C)]. (**F**) Top: Example of walking speed for one fly, showing bouts of activity. Bottom: Probability of walking speed calculated from all data (flies and times) at each of four conditions, showing a bimodal distribution with a large peak in zero that represents inactive flies; dotted lines indicate Gaussian fit $ae^{-{\left(\frac{x-b}{c}\right)}^{2}}$to the walking speed of active flies. (**G** and **H**) Parameter estimates from the Gaussian fit: amplitude (*a*) as an estimate of the activity levels (probability to be active) and mean (*b*) as an estimate of the mean walking speed during active times. Error bars indicate confidence intervals on the fitted parameters.

We next asked whether wiring consequences of developmental temperature affect odor-driven behavior. We tested the odor response of individual, 10-day-old flies, tethered to walk on a spherical treadmill (Fig. 3A). On average, a puff of 2-butanone elicited a stronger increase in walking speed in flies developed at 18°C compared to 25°C (Fig. 3B). These odor responses were highly reproducible across repetitions of the stimulus (Fig. 3D, left), although variable across individuals (Fig. 3D, right). Similar results were obtained with vinegar (fig. S3, A to C). Basal walking speed was, on average, lower for flies developed at 25°C (Fig. 3C). In general, we found no correlation between basal speed and response, as slow and fast walking flies could respond equally well to odors (fig. S3B).

To test whether these findings were specific to this assay and to better understand the effect of temperature on walking behavior, we used a “free-walking” arena. We tracked the flies’ positions while they explored a large circular arena (40 cm in diameter) hiding an odor source in a random position. At all times, more flies raised at 18°C visited the odor source and spent more time at the odor than flies raised at 25°C (Fig. 3E). The difference in behavior between developmental conditions was maximal in the first 7 min of the assay, when the odor landscape was novel. Similar results were obtained with vinegar (fig. S3, D to F) and for a higher testing temperature of 32°C (fig. S3, G and H).

To separate the effect of developmental temperature on baseline walking speed and odor approach, we analyzed data collected in control experiments with no odor. Because flies move in bouts of activity (Fig. 3F, top trace), we asked whether developmental and test temperatures affect how often the fly is walking (activity level) and/or how fast it walks when it is active (mean speed). To assess this, we plotted the probability of walking speed for all conditions tested (Fig. 3F). The peak at zero represents flies that are not walking. The second peak contains all walking flies and can be fitted by a Gaussian. The mean of the Gaussian indicates how fast flies walk when they are moving (mean speed; Fig. 3H). The amplitude of the Gaussian indicates how often the flies are walking instead of standing still (activity level; Fig. 3G). An increase in testing temperature shifts the Gaussian curve right, to higher walking speed, while an increase in developmental temperature slightly lowers the amplitude of the Gaussian, to lower activity. This means that developmental temperature does not affect walking speed but activity level, two parameters that were confounded in the analysis of the ball assay data (Fig. 3C). We conclude that developing flies at 18°C rather than 25°C does not induce major motor deficits but has a slight influence on the propensity of the flies to walk. Because the activity levels of flies developed at 18°C are higher and the walking speed is the same compared to 25°C, the higher odor occupancy of these flies cannot be attributed to inactivity nor to poor locomotion. Together, this argues that approach behavior toward 2-butanone and vinegar is increased when flies develop at lower temperatures.

### The effect of developmental temperature on odor coding

**Fig. 4.**
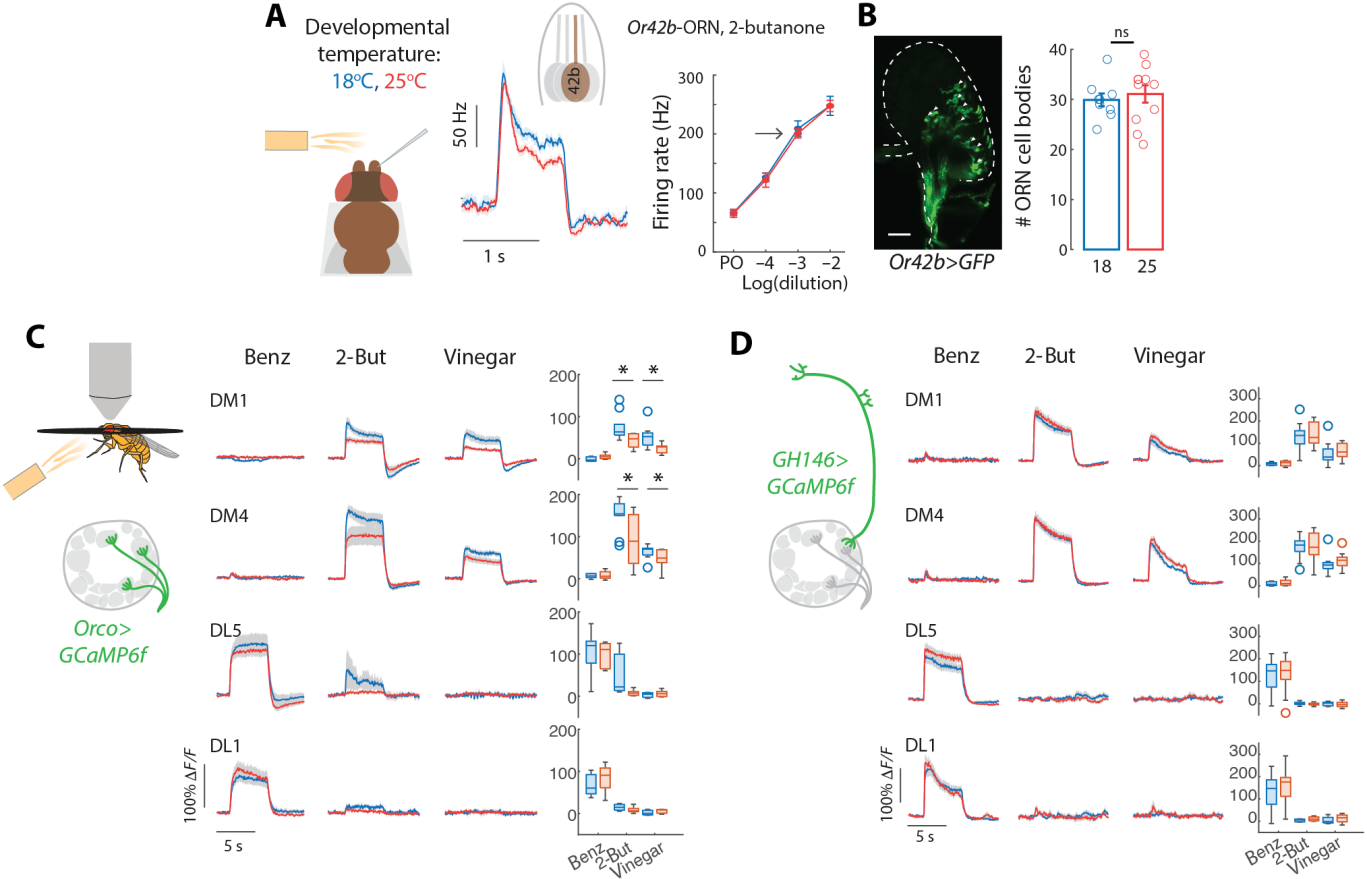
Scaling and normalization of odor responses in input and output neurons of the AL. (**A**) Single-sensillum recordings from the ab1 sensillum containing a single *Or42b*-ORN. Left: Firing rate response to a 1-s pulse of 2-butanone at 10^−3^ dilution calculated in 100-ms sliding window. Right: Mean peak response for each concentration tested [*n* = 10 (18°C) and 11 (25°C)]. (**B**) Confocal image of the antenna showing GFP expression in *Or42b*-ORNs. Bar plot indicates mean and SEM [*n* = 9 (18°C) and 11(25°C)]. (**C**) Calcium imaging from ORN axon terminals in the AL, quantified within four glomeruli for flies developed at the different temperatures [*P < 0.05, *P* value calculated for the average activity during the first second of the stimulus, *n* = 6 (18°C) and 10(25°C)]. (**D**) Same as (C), but the calcium reporter was expressed in uPNs and responses were quantified in the dendritic arborizations within corresponding AL glomeruli, *n* = 16 (18°C) and 18 (25°C). Shaded areas indicate SEM.

Next, we asked whether the enhanced response to appetitive odors is due to altered odor representations in the AL and related to temperature-dependent wiring. Extracellular recordings from single sensilla showed that *Or42b*-ORNs transduction currents (fig. S4A) and firing rates (Fig. 4A) elicited by odor stimuli do not differ in flies developed at different temperatures. Similar results were obtained for *Or59b*-ORNs, innervating glomerulus DM4 (fig. S4, B and C). Moreover, within the antenna, the number of cell bodies labeled by the Or-specific GAL4 line did not differ between the two temperatures (Fig. 4B). We then expressed the calcium sensor GCaMP6f in ORNs (labeled by *Orco-GAL4*) and imaged activity in their axon terminals in the AL in response to odor stimuli. The glomeruli DM1 and DM4 showed higher presynaptic calcium responses in flies developed at 18°C (Fig. 4C). Because the number of action potentials and the number of ORNs did not differ with temperature, we conclude that larger calcium transients in these glomeruli reflect the larger number (and size) of synapses (Fig. 1, I and J). This raises the possibility that uPNs postsynaptic to the *Or42b*- or *Or59b*-ORNs might be activated more strongly by an odor stimulus. Unexpectedly, the odor responses of uPNs in all glomeruli tested were not different in flies developed at 18° and 25°C (Fig. 4D). Similarly, we found that the odor responses of a subset of mPNs—measured from their axonal projections in the LH—were the same in flies developed at the two temperatures, with a significant difference only for benzaldehyde (fig. S4D).

We conclude that odor representations in the output of the AL are robust to changes in developmental temperature, also for the appetitive odors that we tested behaviorally. Innate odor preference is certainly affected by metabolic and feeding state (*38*) and usually so through modulation of olfactory sensory pathways (*39*, *40*). The fact that the odor responses in PNs are the same at the two developmental temperatures indicate that feeding-dependent pathways previously reported to modulate odor preference are not activated, probably because flies are adapted to the same temperature for 10 days before the experiments and are not in an acute feeding deficit.

### Impact of developmental temperature on wiring and function of the LH

Because the measured output activity of the AL is invariant to developmental temperature (Fig. 4), the different behavioral phenotypes observed could result from differences downstream of the AL. Innate odor preference is determined by the wiring of uPNs onto LH neurons (*41*) and modulated by mPNs (*24*–*27*). We therefore asked whether the connections of these cell types onto LHNs were also altered by developmental temperature.

**Fig. 5.**
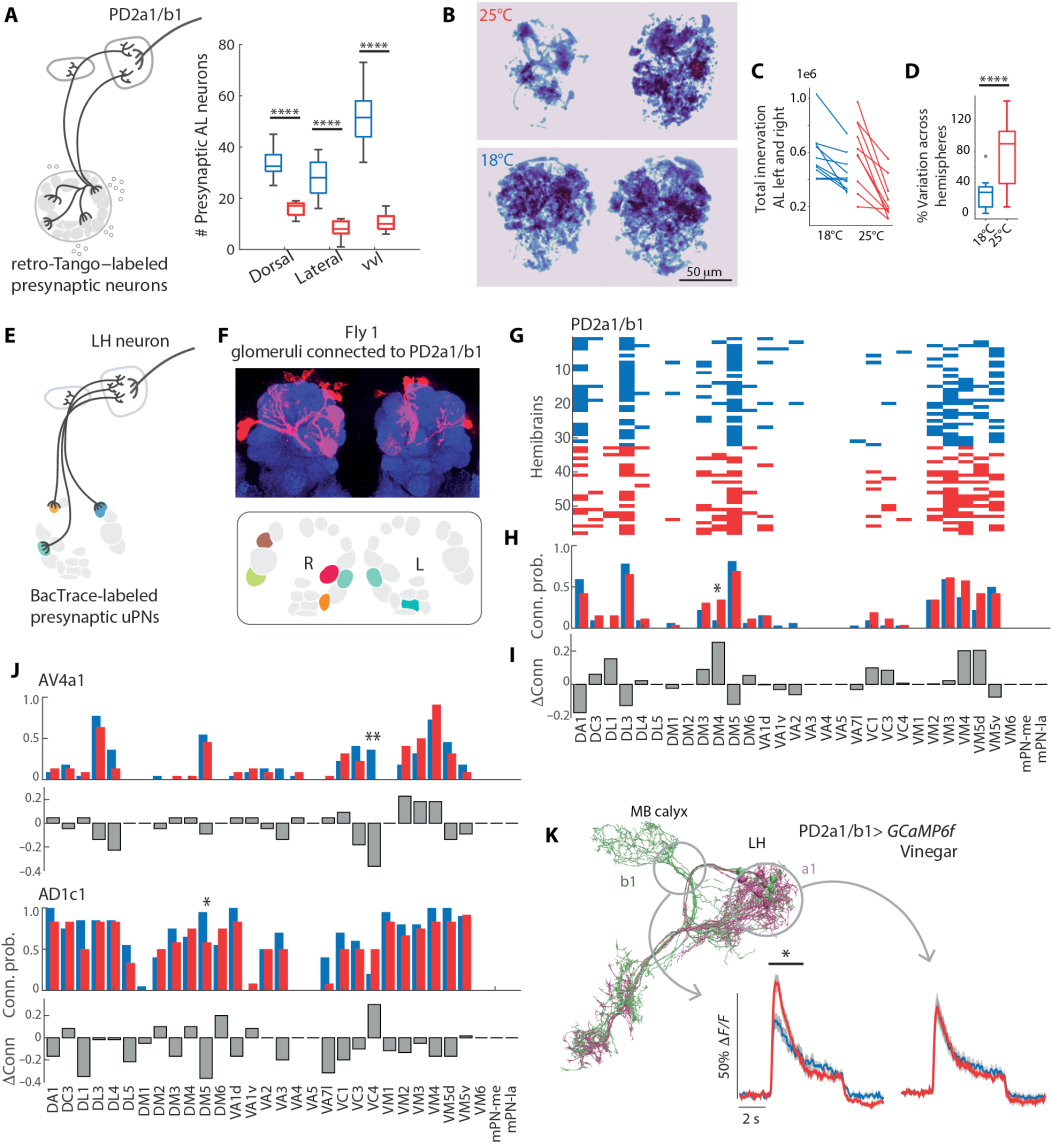
Developmental temperature affects LH neurons’ connectivity to PNs and their odor response. (**A**) Schematics of the *retro*-Tango experiment starting from PD2a1/b1. Boxplot shows median, quartiles, and minimum/maximum values for the number of PD2a1/b1 presynaptic partners from three AL cell clusters; vvl includes cell bodies in the ventral and ventrolateral cluster [*P* < 10^−4^, *n* = 12 (18°C), and 11 (25°C) hemibrains]. (**B**) Example of *retro*-Tango labeling of the AL (Z-projection of the binary mask of fluorescence intensity). (**C**) Quantification of volumes of segmented neurons labeled by *retro*-Tango in L and R hemispheres. Areas are in pixels squared. (**D**) Symmetry in volume innervation calculated as 100 × (volume left − volume right)/(2 × tot volume). *P* < 0.0001, *t* test. (**E**) Schematics of the BAcTrace experiment, showing a LH neuron and candidate presynaptic uPNs from the AL. (**F**) Confocal image of a sample brain showing labeled uPNs arborizations in the AL and binary connectivity patterns of the uPNs (identified by their glomerular innervation) to PD2a1/b1 in a sample brain. (**G**) Connectivity matrix between glomeruli (*x* axis) and PD2a1/b1 in individual hemibrains (*y* axis) in flies developed at 25°C (red) or 18°C (blue). (**H**) Probability to find a certain glomerulus connected to PD2a1/b1 for each temperature. (**I**) Difference in wiring probability (25° to 18°C). *P < 0.05, **P < 0.01, chi-square test, *n* = 32 (18°C) and 26 (25°C). (**J**) Difference in wiring probability for two other LH neuron types, AV4a1: *n* = 12 (18°C) and 20 (25°C); AD1c1: *n* = 10 (18°C) and 20 (25°C). (**K**) Calcium responses of PD2a1/b1 to vinegar in flies developed at 25°C (red) or 18°C (blue). Imaging was performed on two focal planes to capture potential differences between the a1 and b1 subtypes, indicated by circles in the EM skeleton. Shaded areas indicate SEM.

First, we used a pan-neuronal retrograde tracer, *retro*-Tango (*42*), to label all the presynaptic partners of the LHN PD2a1/b1 (Fig. 5A). We chose PD2a1/b1 because it was shown to be functionally connected to DM1 (*43*), although its role in driving innate behavior remains unclear and could be context dependent (*44*–*46*). *Retro*-Tango labels a larger number of presynaptic neurons at the lower developmental temperature, consistent with a general scaling of connectivity throughout the brain. The change in connectivity was larger in the ventral cluster, which contains mPNs (Fig. 5A). An analysis of the AL innervations revealed denser and more symmetric AL innervations of PD2a1/b1 presynaptic partners at 18°C compared to 25°C (Fig. 5, B to D). This is consistent with a more reliable recruitment of mPNs that innervate the whole AL at 18°C. At 25°C mPNs seem to be more stochastically connected to PD2a1/b1 leading to notable asymmetries and patchy innervations of the AL (Fig. 5B and fig. S5A).

In a second approach, we aimed at identifying the glomeruli connected to PD2a1/b1 by uPNs, by using the genetically encoded retrograde tracing tool BAcTrace (*47*) (Fig. 5E). Differently from *retro*-Tango, this tool only labels presynaptic partners from a pool of genetically targeted neurons, in this case 27 uPNs and three mPNs (*VT033006-LexA*). Although the innervation of uPNs’ axons into the LH have been shown to be stereotypic across individuals (*48*), we found a large degree of interindividual variation and asymmetry in connectivity across AL hemispheres (Fig. 5F) [also reported in (*47*)]. An analysis of connection probability shows that developmental temperature influences the connectivity pattern of PD2a1/b1 to AL glomeruli, with a stronger effect in the DM4 glomerulus (Fig. 5, G to I). Temperature, however, does not affect the total number of connected glomeruli (fig. S5, B and C) nor the number of PD2a1/b1 neurons labeled (fig. S5D). Similarly small, but significant shifts in connectivity were observed for two other LHNs, AV4a1 and AD1c1 (Fig. 5J, and fig. S5, E to H).

To link these changes in wiring to function, we performed calcium imaging from PD2a1/b1 neurons. We separated the a1 and b1 subtypes by imaging from two focal planes (Fig. 5K). The b1 subtype showed differences in odor response in flies developed at the two temperatures (Fig. 5K). This demonstrates that PD2b1 function is not robust to developmental temperature, leading to a different LH output in response to odor stimuli. Therefore, while a general scaling of connectivity occurs across the brain, functional consequences are circuit specific and depend on the type of synaptic partners recruited.

## DISCUSSION

**Fig. 6.**
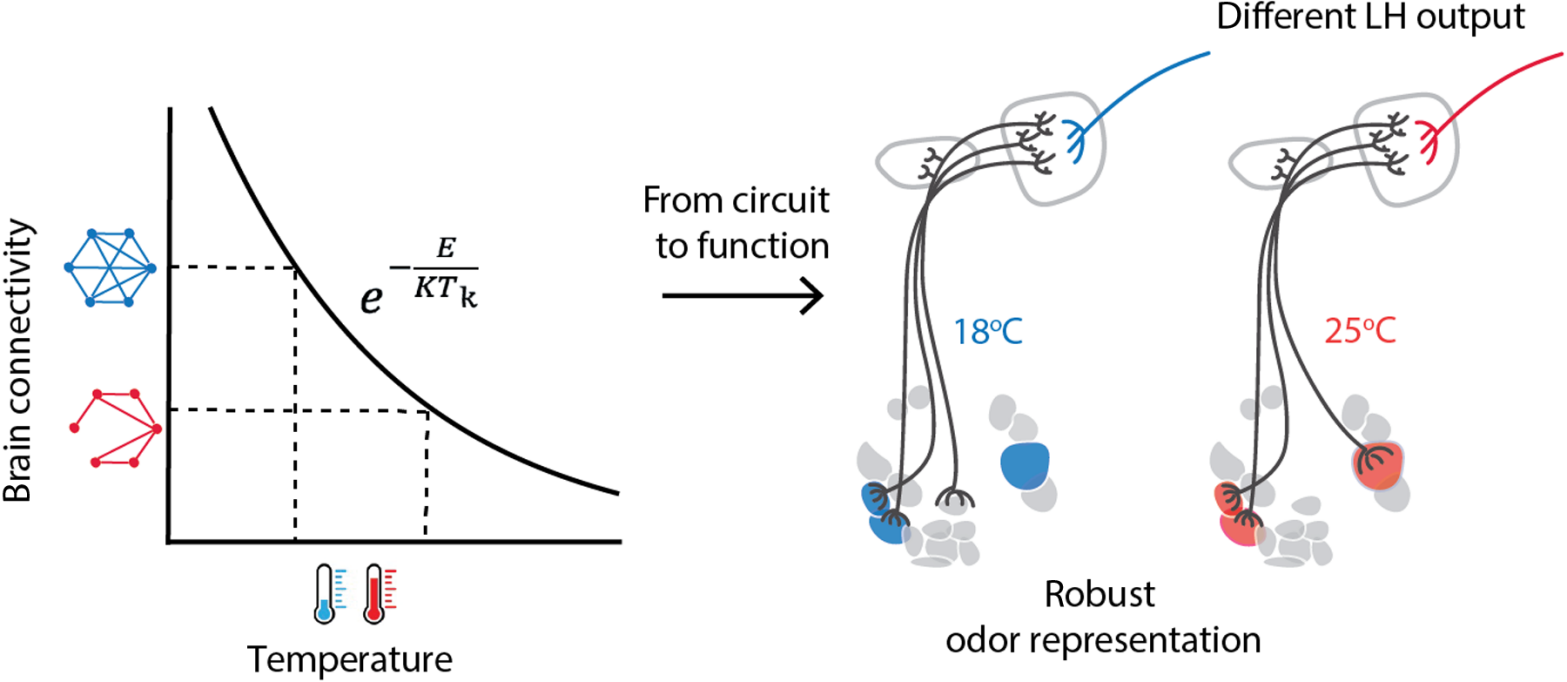
Linking developmental temperature to neural circuit function. Brain connectivity scales inversely to developmental temperature. This general scaling is consistent with a theory that postulates different metabolic constraints on neural and body development. In the AL, odor representations are invariant to the effect of temperature on circuit organization. In the LH, a downstream area that controls innate behavior, developmental temperature affects the olfactory inputs into some LHNs.

The development of a whole organism requires the parallel and coordinated growth of body mass and, in the brain, the establishment of functional synaptic connections. Developmental programs are highly temporally structured, raising the question of how they are affected by a temperature-dependent change in developmental speed. Here, we propose that if developmental speed is constrained by varying metabolic reactions across cell types, then temperature manipulation could potentially disrupt the temporal coordination of their developmental programs. We demonstrate that this temporal mismatch can provide an explanation for the change in neural growth and connectivity induced by developmental temperature (Fig. 6, left). Developmental temperature has consequences on odor-driven behavior, but we show that this is not due to different odor responses or sensitivity across the olfactory glomeruli. Therefore, despite a scaling in connectivity, odor encoding is robust to developmental temperature. Odor information, however, converges differently onto LH neurons, with possible consequences for behavior (Fig. 6, right).

### Metabolic constraints on the development of the neural system

Through an analysis of filopodia dynamics, Kiral *et al.* (*16*) had proposed that an increased number of synapses at lower developmental temperature is the result of a difference between the scaling of neural and animal growth rates. Here, we have formalized this hypothesis in a mathematical model of growth. We show that fly developmental times scale exponentially with temperature in agreement with proposed theories that assume a metabolic constraint to growth rate. We then extended this theoretical framework to model the rate of neural growth and synapse formation, with two main assumptions. The first is that neural/synaptic growth scales with the fractional power *z* = ^3^/_4_. This value was previously justified starting from geometrical considerations on the fractal nature of the distributions of resources throughout capillaries in a 3D volume (*34*). Our data at fixed temperatures are consistent with any *z* < 1, but the developmental outcome measured in presence of cycling temperatures is well predicted by a ^3^/_4_ exponent (*49*). Nonetheless, this scaling law requires further experimental confirmation in the neural system.

The second assumption we made is that the pace of ontogenesis (whole animal development) and neural development are limited by different metabolic reactions. We currently have no direct evidence to support this hypothesis. However, recent works have linked developmental pace to metabolic rates. For example, species-specific developmental rates have been associated to differences in mitochondrial metabolism (*50*, *51*) and manipulations of metabolism directly scale developmental rates (*51*). An intriguing possibility is that these metabolic processes may vary across cell types, particularly in neurons, though this remains to be clarified (*52*). Recent analysis of single-cell transcriptomes in zebrafish demonstrated that developmental temperature has cell type–specific effects on proteostasis (*53*) with possible consequences on developmental pace. Future studies will have to clarify whether differences in cell metabolism determine the effect of temperature on brain wiring. In the meantime, the role of metabolism in this context remains an assumption.

### Mechanisms for robust function in a synaptically enriched circuit

The analysis of neural connectivity of distinct glomeruli reveals that lower developmental temperature leads to an overall increase in synaptic partners, as well as a larger number and size of synapses (fig. S6A). This synaptic scaling is consistent with the larger calcium transients measured in some of the glomeruli, in the corresponding ORN axons (fig. S6A). Because ORNs are cholinergic, this increased presynaptic activity could lead to higher excitatory responses in postsynaptic PNs that send odor information to higher brain areas. Unexpectedly, this was not the case, and the responses of PNs was mostly invariant for the two temperatures considered (fig. S6, B and C). This compensation might rely on inhibitory LNs that are recruited in larger numbers at lower temperatures. The balance between excitation and inhibition is key for the function of healthy brains, and there is evidence that this balance is achieved developmentally during synapse formation (*54*). In the fly olfactory system, γ-aminobutyric acid (GABA) mediated inhibition is distributed at both pre- and postsynapses in ORN-uPN connections (*55*, *56*). Presynaptic inhibition could therefore explain why in some glomeruli (DL1 and DL5) calcium transients are already compensated at the ORN axon terminals (fig. S6A), although this remains to be confirmed experimentally. Last, inhibition might not be the sole mechanism that keeps responses temperature invariant. It is possible that the presynaptic scaling is also a homeostatic response to weaker postsynapses (*57*, *58*) or lower release probability (*59*). Overall, we conclude that within a range of temperatures, the peripheral olfactory system is designed to compensate for the developmental effects on circuit wiring and to keep odor information invariant to this environmental factor.

### Circuits for the modulation of odor preference downstream of the AL

Despite the robust odor representations within the AL, behavioral responses to appetitive odor cues are strongly affected by developmental temperature in two different assays (Fig. 3). This indicates that the scaling in brain connectivity is not fully functionally compensated, and the odor induced activity in circuits downstream of the AL is dependent on developmental temperature. Innate odor preference arises in the LH, where PNs target LHNs within a complex wiring logic. Here, LHNs pull information from subsets of glomeruli with ecologically related odor response profiles (*43*, *44*). Our analysis demonstrates that the odor information received by the LH is temperature dependent because of small but significant differential wiring of PNs (fig. S6D). PD2b1 neurons have different odor responses in flies developed at different temperatures, and therefore, LH output is not invariant to developmental temperature.

These changes in connectivity and function are likely only a lower bound on the effect of developmental temperature. First, only half of the uPN types was included in our analysis, and we might have neglected more relevant connections. Second, the connectivity and odor responses of other LHNs might be affected. PD2a1/b1 neurons are required for memory retrieval (*45*), but their role in innate behavior is likely context dependent (*46*). They might be involved in approach toward appetitive cues, although their activation is not sufficient to drive behavior (*44*). Most likely, PD2a1/b1 neurons act synergistically or redundantly with other LHNs with temperature-dependent wiring, although the logic of these circuits remains to be understood.

Last, odor information is transmitted to higher brain areas not only by uPNs but also by mPNs that target the LH and the protocerebrum. The connectivity of mPNs to ORNs and LHNs is also affected by temperature (fig. S6, C and D). The function of this large population of neurons (>50% of the total number of PNs), however, is still unclear. Half of mPNs are predicted to be GABAergic (*23*): These modulate odor preference and discrimination (*24*, *26*, *27*), and silencing inhibitory mPNs reduces approach toward appetitive odors (*24*). The other half of mPNs is predicted to be cholinergic (*23*), with a still unknown contribution to both downstream odor processing and behavior. Therefore, further understanding of mPN function and LH circuit organization is required to link the temperature-dependent plasticity to the behavior we observe.

### Linking temperature-dependent wiring to behavior: Current limitations

The observation that developmental temperature scales connectivity throughout the brain presents a substantial challenge in establishing a causal link between circuit architecture and behavior. We were able to exclude the early olfactory circuit as the origin of the observed behavioral differences. However, we cannot conclusively demonstrate that these differences arise from changes in the output of the LH, as other downstream pathways could also be affected by developmental temperature. Therefore, the relationship between LH output and behavior in flies developed at different temperatures must be regarded as correlative. Overall, our analysis shows that predicting circuit function—whether in terms of neural activity or behavioral outcomes—based on circuit connectivity remains a challenge in the context of these developmental manipulations.

### Phenotypic variation and constrained pathways

Overall, our study suggests that the effect of developmental temperature on neural circuit wiring and function is heterogeneous across circuits and likely depends on the spatiotemporal availability of synaptic partners (*1*). Clearly, some connections in the brain are critical for survival, and these have probably evolved to correctly wire and function no matter the temperature. Developing flies on extremely high temperatures revealed what is considered to be the backbone of the olfactory pathway, i.e., the wiring of ORNs onto a single uPN and a few mPNs and LNs (glomerulus DM1, Fig. 2B). Studying these extreme conditions might provide insights on evolutionary constraints on circuit design.

The brain has evolved many strategies to keep circuits’ function robust to environmental factors (*60*, *61*). While such robustness holds true within some subcircuits (individual glomeruli in this study), it cannot be assumed to occur throughout the brain (LH output in this study). In this way, environmental factors can lead to observable variation in behavioral phenotypes. Our study raises the question of whether such variation in brain wiring is an evolutionary selected feature for adaptation to the environment, posing new challenges for understanding brain development in poikilothermic animals.

## MATERIALS AND METHODS

### Experimental model and fly husbandry

Flies were raised on standard molasses-based food, at 65% humidity and on controlled 12-hour:12-hour light-dark cycles. All flies were kept at 25°C as embryos and after eclosion. Flies were placed at different temperatures (12°, 18°, 25°, and 31°C depending on the experiment) between the third-instar larva stage (L3) and the end of metamorphosis. As the time of development depends on temperature, we call the end point of metamorphosis P-100%. For optogenetics experiments, flies were kept in standard molasses-based food with 1 mM all-trans retinal (Sigma-Aldrich) in the dark for ≥72 hours before experiments. In all experiments, only females were used. Exact genotypes are given in tables S1 and S2.

### Temperature cycle experiment

For temperature cycle experiments, flies were kept on 12-hour:12-hour light-dark cycles. Temperature varied in parallel to light with the high temperature corresponding to the light cycle. The temperature ranges (minimum to maximum) used were 20° to 28°C, 18° to 30°C, 16° to 32°C, and 14° to 34°C. The maximum temperatures were held for 8 hours and shifted to the minimum temperature gradually in 4 hours.

### Immunohistochemistry and confocal

Female flies (9 to 11 days after eclosion) were anesthetized with ice and then briefly submerged in ethanol 70%. Flies were dissected on cold phosphate-buffered saline (PBS) for no longer than 20 min and fixated for 50 min in 2% paraformaldehyde (Polysciences, diluted in PBS) rotating at room temperature. All subsequent incubation and washes were done while rotating, in the dark. Brains were washed three times in PBT (PBS with 0.5% Triton X-100, Roth) for 15 min and then blocked for 1 hour in 5% normal goat serum (Thermo Fisher Scientific, in 0.3% PBT). Samples were incubated in primary antibody mixture (chicken anti-GFP 1:1000; rabbit anti-DsRed 1:500; mouse anti-nc82 1:25) for 48 hours at 4°C, then washed in PBT (3× of 15 min) and incubated in secondary antibody mix (goat anti-chicken Alexa Fluor 488; donkey anti-rabbit Alexa Fluor 568; donkey anti-mouse Alexa Fluor 647, all at 1:200) for 48 hours at 4°C. Last, brains were washed three times in PBT and mounted in VectaShield (Biozol). Brains were imaged on a Leica SP8 microscope with a 20×, 40×, or 63× objective depending on the experiment. After image acquisition, the number cell bodies of postsynaptic partners were manually counted using Fiji’s cell counter plugin. Cell body numbers were classified according to the position around the AL: dorsal, lateral, or ventrolateral (including both ventrolateral and ventral clusters). For reagents, see table S1.

#### Brp^Short^ analysis

Confocal images of individual DM1 glomeruli were processed using a custom code in Python, with the package pyclesperanto (*62*). Images contained a Brp^Short^ (GFP) and Brp (nc82) channel. Both channels were preprocessed with Gaussian blur (1.0, 1.0, 1.0) and top hat box (20.0, 20.0, 1.0). The DM1-ORN mask was made using the Brp^Short^ channel, by applying Voronoi Otsu Labeling and then merging the touching labels. For the whole DM1 glomerulus mask, the DM1-ORN volume was closed using the function closing labels. Single nc82-labelled puncta were segmented by Voronoi Otsu Labeling and restricted to the DM1 glomerulus mask. The volume and fluorescence intensity of labeled regions were acquired using the statistics of labeled pixels in pyclesperanto.

#### *Retro*-Tango quantification

As for Brp^Short^ analysis, *retro*-Tango confocal stacks were processed using a custom Python script. For each AL, a manual mask was done to delimit the region of interest and crop the original images. Presynaptic neurons were preprocessed by applying Gaussian blur (1.0, 1.0, 1.0) followed by top hat box (10.0, 10.0, 1.0). Presynaptic neurons were then binarized with Gauss Otsu Labeling. Volumes of labeled regions were acquired using the statistics of labeled pixels (pyclesperanto).

### In vivo calcium imaging

Flies were developed at either 18° or 25°C. At 9 to 11 days after eclosion, flies were anesthetized on ice and mounted on a custom holder using ultraviolet (UV)–cured glue (Bondic). Saline solution [5 mM Hepes, 130 mM NaCl, 5 mM KCl, 2 mM MgCl_2_, 2 mM CaCl_2_, and 36 mM saccharose (pH 7.3)] was added. The cuticle covering the fly’s head, as well as obstructing trachea, were removed. Functional imaging was done on an Investigator two-photon microscope (Bruker) coupled to a tunable laser (Spectraphysics Insight DS+) with a 25×/1.1 water-immersion objective (Nikon). Laser excitation was tuned to 920 nm, and less than 20 mW of excitation was delivered to the specimen. Emitted light passed through a SP680 short-pass filter, a 560 lpxr dichroic filter, and a 525/70 filter. PMT gain was set to 850 V. The microscope was controlled with the PrairieView (5.4) software.

#### Optogenetics

For optogenetic activation light from a 625-nm light-emitting diode (LED) was directed using an optic fiber to the fly’s antenna. The LED was controlled in flight-back mode from the imaging software using an Arduino board, allowing simultaneous acquisition and excitation. The light stimulus protocol consisted of 5-s series of light pulses, presented five times with intervals of 30 s. Stimulus intensity in fig. S1A was measured at the fly position with this protocol.

#### Odor delivery

Flies were exposed to a continuous clean air airflow (1 liter/min), in which either an odor stream (100 ml/min) or a clean balancer airflow (100 ml/min) was redirected through a solenoid valve (LEE), so that the final airflow reaching the fly was around 1.1 liters/min. For creating the gas dilutions, four mass flow controllers were used (Analyt-MTC) and controlled using a custom MATLAB (MathWorks) script and an Arduino board. Odors were prepared as a liquid 5-ml 10^−2^ volumetric dilution in 20-ml glass vials (2-butanone and benzaldehyde in mineral oil and apple cider vinegar in MiliQ Water). The final volumetric gas dilution used was 10^−5^. Odor stimulation consisted of three repetitions of a 5 s, with 30-s intervals in between. For chemicals, see table S1.

### Electrophysiology

Single-sensillum recordings were performed as previously described (*63*) using a silver chloride electrode and glass pipettes filled with sensillum lymph ringer. Electrical signals were amplified using an extracellular amplifier (EXT-02F-1, npi) with head stage (EXT-EH), band-pass filtered (300 to 5000 Hz), and digitized at 20 KHz using a NI board (NI-6212). Data were acquired with the matlab toolbox kontroller (*64*) (https://github.com/emonetlab/kontroller). Spikes were sorted using a custom MATLAB routine available at https://gitlab.rlp.net/mrtlllab/zuefle_batista_etal_2024.

#### Odor delivery

Flies were exposed to a constant airflow (1 liter/min), and an odor stimulus was delivered by switching a three-way solenoid valve that directed a secondary airflow (100 ml/min) through a Pasteur pipette as in (*63*, *65*). The pipette contained a filter paper with 50-ml odor dilution. Volumetric odor dilutions were prepared in either mineral oil or MiliQ Water. Stimuli were controlled by custom made software in MATLAB and Arduino.

### Behavioral experiments

#### Spherical treadmill

Experiments were conducted at 32°C in a closed custom arena. The spherical treadmill consisted of a 15-mm-diameter polyurethane foam sphere (FR-7120 foam, General Plastics) floating on an air column. The sphere was coated with two layers of classic wood glue (Ponal, 25% in water), and then a random nonuniform pattern was drawn using two layers of acrylic black paint (Black 3.0, Culture Hustle). All coats of paint were allowed to dry overnight. The odor delivery system was similar to the one described above for in vivo calcium imaging experiments but with a differing airflow rate controlled by Alicat Scientific MFCs. Continuous clean airflow was 90 ml/min, and both the odor and balancer airflows were 10 ml/min. Videos were acquired with a XIMEA xiQ video camera, placed 10 cm from the treadmill. The treadmill ball was illuminated by a panel of 940-nm LEDs (Solarox) and an extra LED on the air column was visible in the video and turned on simultaneously to the odor stimulus to trigger the data.

*Experimental protocol*. For experiments, 9- to 10-day-old female flies were cold anesthetized and secured to a needle at their thorax on the dorsal side using a UV-hardening glue (Bondic) and positioned on the sphere with the help of a 3D micromanipulator. Before recording, flies were acclimatized to walking on the sphere for 10 to 15 min with no stimulus being presented. Subsequently, video recording and the odor stimulation were started. Videos were acquired using the XIMEA CamTool software: The exposure was set to 10,725 ms, the gain to 2.6 Db, and the frame rate to 80 fps. The odor stimulation was controlled through MATLAB by an Arduino UNO Rev3 and consisted of at least 19 repetitions of 5-s-long odor stimuli and 20-s-long interval without odor.

*Video processing and analysis*. Fly moving speed was calculated a posteriori from the video recordings using the open-source software library FicTrac (*66*), which provided direction and walking speed of the animal for each frame in the video. The video recordings were also analyzed in MATLAB to extract the stimulus trigger from the LED placed in the field of view. Last, the output data from Fictrac and the time points obtained from the video were analyzed in MATLAB so that the moving speed during and outside odor presentation could be quantified. Flies that had a basal walking speed lower than 2 mm/s were discarded.

#### Free-walking assay

A free walking area was contained in a thermally controlled black box (100 cm by 45 cm by 45cm) shielded from room light, fully closed with a frontal door, and equipped with a heating system and thermostat (H-TRONIC GmbH, Product ID: 1114430). The box was heated up by an air stream created by a fan and homogeneously distributed by a diffuser. A blue LED stripe (470 nm, Paulmann Licht GmbH, product ID: 78979) was positioned around the walking arena to ensure stable illumination during experiments. Videos were recorded with a Basler Camera (Basler acA2040-90um) placed on the ceiling of the box and equipped with f12mm lens (Basler C10-1214-2 M-S), using Pylon viewer (64-Bit, 6.3.0.10295). The walking arena had 40.2-cm diameter and 2-cm height and was composed of four stacked layers and three overlapping plates (glass or plexiglass). The bottom layer contained six holes, arranged at the corners of a hexagon, where 1.5-ml glass vials (Fisherbrand 11565874) containing the test odor could be screwed in. On top of this, a Teflon-coated porous sheet (FIBERFLON GmbH & Co. KG, product ID: 408.07 P) provides a walking surface for the flies hiding the odor location. The mid-arena layer consists in a sloped (at 11°, 5-cm length) ring that defines the accessible walking area. To seal the walking arena, we used a glass plate coated with Sigmacote (Sigma-Aldrich Co.) to prevent fly walking upside down. This behavioral setup was built by the workshop of the Biology Department at Johannes Gutenberg Universität Mainz.

*Experimental protocol*. We tested female flies developed at 18° or 25°C, 5 to 7 days after eclosion. One hour before the experiment, flies were transferred into a vial with only a small piece of filter paper soaked in water and kept at room temperature. For experiments carried at 32°C, the fly vials were incubated for 15 min in a 32°C water bath. To create an odor gradient inside the arena, 5 min before the start of each experiment, a 1.5-ml glass vial containing 1 ml of test odor was placed in one of the six possible odor positions in the behavioral setup. For each trial, a fresh odor vial was used, and the position was pseudo-randomized. Each trial consisted of 10 to 15 female flies exposed to either apple cider vinegar (10^−2^ in MilliQ water) or 2-butanone (10^−2^ in mineral oil) or tested with empty vials. Flies were gently pushed inside the arena using a custom fly transfer tube and the recording was immediately started. All experimental videos were recorded at 20 fps for 15 min and saved in mp4 format. At the end of each trial, the flies were removed and discarded. The initial condition was restored by removing the odor vial and the cover glass plate, replacing the Teflon sheet with a clean one, and letting the whole system ventilate for 5 min.

*Video processing and analysis*. All required steps to preprocess the raw videos were done using the Python 3.9.12 distribution ANACONDA (version 4.13.0). Scripts were written using Virtual Studio Code (version 1.81.1). Recorded mp4 videos were processed and video tracked with the software TRex (version 1.1.8_3) (*67*). The output files were analyzed using custom Python and MATLAB scripts available at https://gitlab.rlp.net/mrtlllab/zuefle_batista_etal_2024. A threshold of 5 cm was chosen to determine whether the fly had located the odor source; variations of this threshold do not alter the results. Odor occupancy was calculated as the integral under the curve in the first 7 min, being 1 if all flies spend 100% of the time at the odor.

### Connectome analysis

We used the Hemibrain dataset (hemibrain:v.1.2.1) (*20*). For Fig. 1 (K and L) and fig. S1 (E and F), we considered all synapses from the *Or42b*-ORNs of the left and right antennae within the DM1 glomerulus of the right hemisphere, which were selected by clustering the synapses on the basis of their 3D coordinates. The analysis was restricted to synapses of the right hemisphere, as postsynaptic partners are fully reconstructed only on this side. We calculated the number of synapses between each ORN and each postsynaptic neuron. Connections with less than three synapses from a single ORN were discarded. Moreover, postsynaptic neurons that received less than 10 total synapses were discarded. The number of synapses was normalized to the total number for this ORN type. Postsynaptic neurons were sorted on the basis of the number of synaptic inputs they received. The same procedure was used for DL1 and DL5. The percentages of LNs and mPNs in fig. S1F are lower bounds, as calculated from the available annotations.

### Theory

To model ontogenetic growth, we follow the same approach of (*3*). We assume that growth scales as the three-fourth power of the mass m [see Discussion and (*34*)] following the equation

$$\frac{\mathrm{dm}}{\mathrm{dt}}=am^{\frac{3}{4}}\left[1-{\left(\frac{m}{M}\right)}^{\frac{1}{4}}\right]$$ (1)

where *M* is the asymptotic mass and *a* is proportional to metabolic rate

$$a=\frac{B_{0}m_{c}}{E_{c}}$$ (2)

with *m*_c_ the cell mass, $E_{c}$ the energy per cell, and $B_{0}$ is the normalization factor of the metabolic rate $B=B_{0}m^{\frac{3}{4}}$ that scales proportionally to the Boltzmann factor: $B_{0}\propto e^{-\frac{E}{KT_{k}}}$ ($T_{k}$ is the temperature in Kelvin, K the Boltzmann constant, and E the activation energy). Therefore, $a\propto e^{-\frac{E}{KT_{k}}}$. Following (*3*), we calculate a with respect to a reference temperature (the water freezing point *T_a_* = 273 K), and replacing $=T_{k}-T_{a}$, we obtain

a=a(T,E)=a(Ta,E)eEKTa2(T1+TTa)≈a(Ta,E)eEKTa2T (3)

where now the temperature *T* is in degrees Celsius. The last approximation takes in account the fact that the relevant temperatures do not exceed 32°C; therefore, TTa is at most 0.1. This approximation leads to an error of about 10% on the exponential fits, but the quality of the model prediction remains unchanged. We keep the approximation for simplicity in the following calculations.

To find the relationship between developmental time and temperature, we integrate the growth Eq. 1 for m≪M (but see below)

$$\int_{0}^{m\left(t\right)}m^{-\frac{3}{4}}\;\mathrm{dm}=\int_{0}^{t}a\left(T,E\right)\mathrm{dt}⟹4m^{\frac{1}{4}}=a\left(T,E\right)\cdot t$$ (4)

Equations 3 and 4 lead to the exponential relationship between developmental time *t* and temperature *T* proposed in (*3*)

$$t=\frac{4m^{\frac{1}{4}}}{a\left(T_{a},E\right)}e^{-\frac{E}{KT_{a}^{2}}T}$$ (5)

We calculate the fold change with respect to a reference temperature *T*_0_ = 25°C by assuming that development results in the same final mass

$$\frac{t}{t_{0}}=e^{-\frac{E}{KT_{a}^{2}}\left(T-T_{0}\right)}=e^{-\alpha \left(T-T_{0}\right)}$$ (6)

with $=\frac{E}{KT_{a}^{2}}$. We use Eq. 6 to fit developmental times in Fig. 2K. This result remains the same if we use the general solution of Eq. 1 from (*3*) (relaxing the assumption ≪M) or if we integrate it from $m\left(0\right)=m_{i}$ instead of m(0)=0.

We now assume that developmental time follows the exponential relationship in Eq. 6, while the wiring of the neural circuit is constrained by a different reaction rate with *E*′ < *E*. In modeling the growth of the neural system, we use *n* instead of the mass *m*, which can be intended as number of synapses, synaptic partners, or axonal branching. *n* follows a similar equation as Eq. 5 leading to

$$\frac{t}{t_{0}}={\left(\frac{n}{n_{0}}\right)}^{\frac{1}{4}}e^{-\frac{E^{\prime }}{KT_{a}^{2}}\left(T-T_{0}\right)}$$ (7)

This results from an initial condition n(0)=0, which is reasonable given the major pruning and regrowth of axons that happens during metamorphosis. Using Eqs. 6 and 7

$$\frac{n}{n_{0}}=e^{-4\frac{E-E^{\prime }}{KT_{a}^{2}}\left(T-T_{0}\right)}$$ (8)

where we define $\beta =4\frac{E-E\prime }{KT_{a}^{2}}=4\frac{∆E}{KT_{a}^{2}}$. If ∆E=0, then there should be no change in the number of synaptic partners. Also note that β can be larger than α ($⟹E>\frac{4}{3}E\prime$) or smaller than α ($⟹E\prime <E<\frac{4}{3}E\prime$). We use Eq. 8 to fit fold changes in the number of synaptic partners in Fig. 2G.

Next, we calculate the developmental time of flies on temperature cycles with maximum and minimum temperatures $T_{1}$ and $T_{2}$. Here, we simplify the cycling temperature protocol to step changes such that the final mass on temperature cycles results from development that occurs half of the time at $T_{1}$ and half at $T_{12}$

$$\int_{0}^{m\left(\tilde{t}\right)}m^{-\frac{3}{4}}\;\mathrm{dm}=\int_{0}^{\tilde{\frac{t}{2}}}a\left(T_{1},E\right)\mathrm{dt}+\int_{0}^{\tilde{\frac{t}{2}}}a\left(T_{2},E\right)\mathrm{dt}$$ (9)

$$⟹m^{\frac{1}{4}}=\tilde{\frac{t}{8}}\left[a\left(T_{1},E\right)+a\left(T_{2},E\right)\right]$$ (10)

Here, $\tilde{t}$ indicates the developmental time on temperature cycles. Assuming an equal final mass, the fold change with respect to a fix temperature $\bar{T}$ is

$$\tilde{\frac{t}{\bar{t}}}=2\frac{a\left(\bar{T},E\right)}{a\left(T_{1},E\right)+a\left(T_{2},E\right)}=2\frac{1}{e^{-\alpha \left(\bar{T}-T_{1}\right)}+e^{-\alpha \left(\bar{T}-T_{2}\right)}}$$ (11)

To calculate the fold change in synaptic connectivity, we use the same logic as before to derive

$${\left(\tilde{\frac{n}{\bar{n}}}\right)}^{\frac{1}{4}}=\frac{1}{2}\tilde{\frac{t}{\bar{t}}}\frac{a\left(T_{1},E\prime \right)+a\left(T_{2},E\prime \right)}{a\left(\bar{T},E\prime \right)}$$ (12)

Moreover, using Eq. 11

$$\tilde{\frac{n}{\bar{n}}}=e^{\beta \bar{T}}{\left(\frac{e^{\gamma T_{1}}+e^{\gamma T_{2}}}{e^{\alpha T_{1}}+e^{\alpha T_{2}}}\right)}^{4}$$ (13)

with $=\frac{E\prime }{KT_{a}^{2}}=\alpha -\frac{\beta }{4}$. We use Eq. 13 to predict the number of synaptic partners in flies developed on periodic temperature cycles in Fig. 2Q. The solutions in Eqs. 11 and 13 further simplify, if we take the mean temperature as the reference temperature $\bar{T}=\frac{T_{1}+T_{2}}{2}$ and $∆T=\frac{T_{2}-T_{1}}{2}$ that is

$$\tilde{\frac{t}{\bar{t}}}=\frac{1}{\text{cosh}\left(\alpha ∆T\right)}$$ (14)

and

$$\tilde{\frac{n}{\bar{n}}}={\left[\frac{\text{cosh}\left(\gamma ∆T\right)}{\text{cosh}\left(\alpha ∆T\right)}\right]}^{4}$$ (15)

which shows that the fold change in developmental time and connectivity scale inversely with the amplitude of the temperature cycles (as γ<α).
