# Supplementary material for: Impact of developmental temperature on neural growth, connectivity, and function
Source: Sci Adv. 2025 Jan 15;11(3):eadp9587. doi: 10.1126/sciadv.adp9587 (PMC11734716; doi:10.1126/sciadv.adp9587)
Supplement: Supplementary file 1 — Figs. S1 to S6 Tables S1 and S2 [file sciadv.adp9587_sm.pdf]

Science Advances
AAAS

Supplementary Materials for

Impact of developmental temperature on neural growth, connectivity, and function

Pascal Züfle *et al.*

Corresponding author: Carlotta Martelli, cmartell@uni-mainz.de

Sci. Adv. **11**, eadp9587 (2025)
DOI: 10.1126/sciadv.adp9587

This PDF file includes:

Figs. S1 to S6
Tables S1 and S2

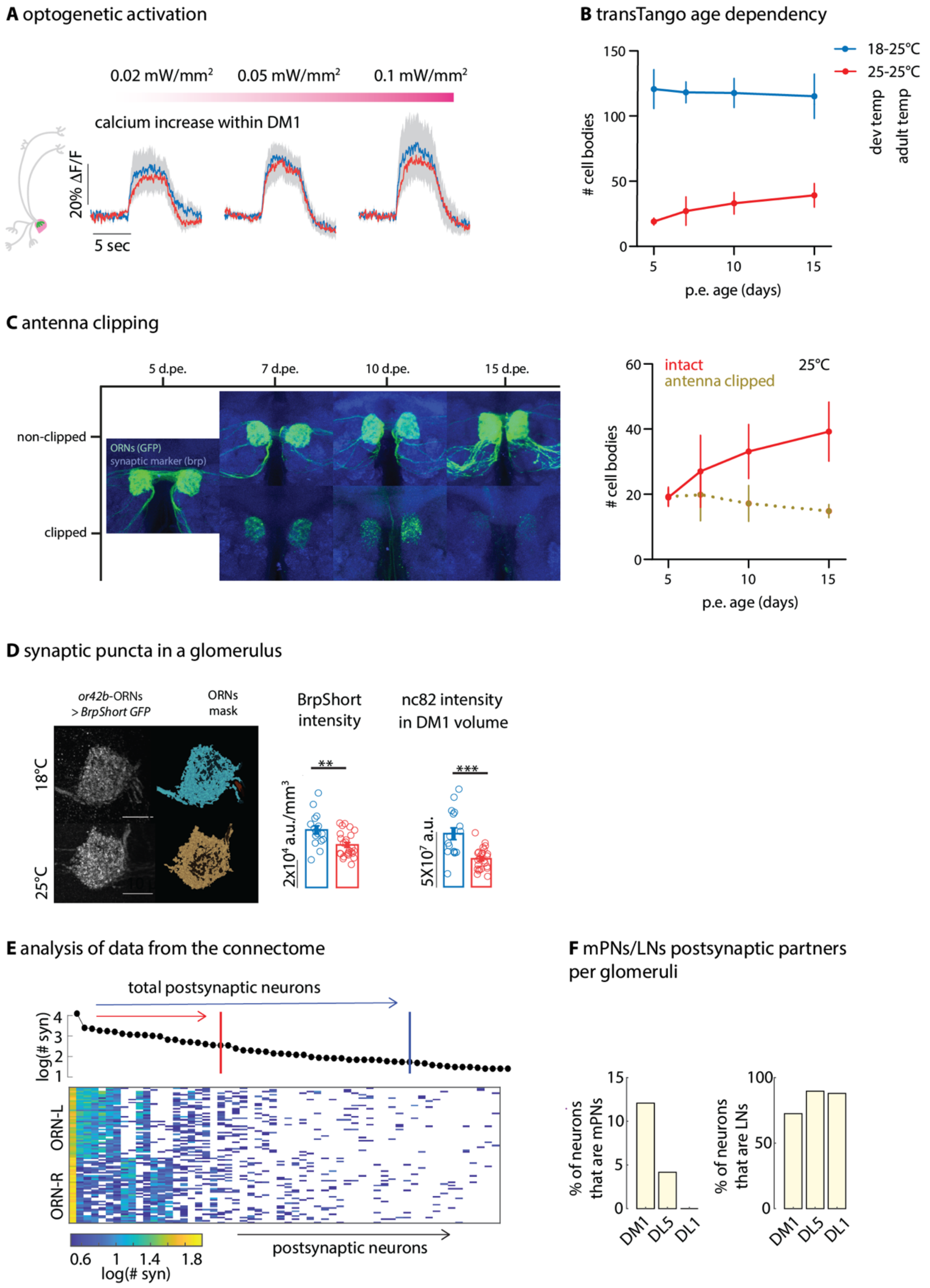

Fig. S1. Tool validation and anatomical quantifications. (A) Optogenetic activation of *Or42b*-ORNs in flies developed at different temperatures. The usual trans-Tango reporter dsRed was replaced by GCaMP6s, and activity of presynaptic neurons induced by CsChrimson. Mean optogenetic response of DM1-postsynaptic partners was quantified in the glomerulus for three stimuli intensities. **(B)** Mean number of cell bodies in the AL labeled by trans-Tango as post-

synaptic partners of *Or42b*-ORNs measured at different ages after eclosion. Error bars indicate standard deviation (N = 5-10). **(C)** Sample confocal images showing the axons of *Or42b*-ORNs across days post eclosion in control flies (top) and in flies whose antenna were clipped on day 5 (bottom). The decrease in GFP indicates axon degeneration. Right: mean number of cell bodies in the AL labeled by transTango as post-synaptic partners of *Or42b*-ORNs measured at different ages after eclosion in control and antenna-clipped flies (N = 8-12). Overall, these experiments indicate that the transTango reporter accumulates over time, nonetheless it is able to detect changes in synaptic connectivity that occur in the adult. The response time could be somewhat slow. (**D)** Left to right: $Brp^{[Short]}$-GFP fluorescence expressed in *Or42b*-ORNs and glomerulus 3D mask reconstructed from it for the two temperatures. Quantification of $Brp^{[Short]}$-GFP and nc82 puncta intensity within the whole glomerulus volume (error bars indicate SEM, n = 16 hemibrains at 18°C and n = 25 at 25°C, Kruskal-Wallis test, $**p<10^{-2}$, $***p<10^{-3}$). **(E)** Bottom: heatmap of the connectivity matrix from hemibrain EM data illustrating the number of synapses made by DM1-ORNs from both antennae on postsynaptic partners in the right hemisphere. Post-synaptic partners on the x-axis are ranked by the total number of input synapses from these ORNs, as quantified in the top plot. Color scale indicates the number of synapses in log scale. The red and blue bar indicate the mean number of postsynaptic partners counted within one hemisphere in the trans-Tango experiment. **(F)** Percentage of postsynaptic neurons that have been annotated in the Hemibrain dataset as multiglomerular projection neurons (mPNs, left) and local neurons (LNs, right).

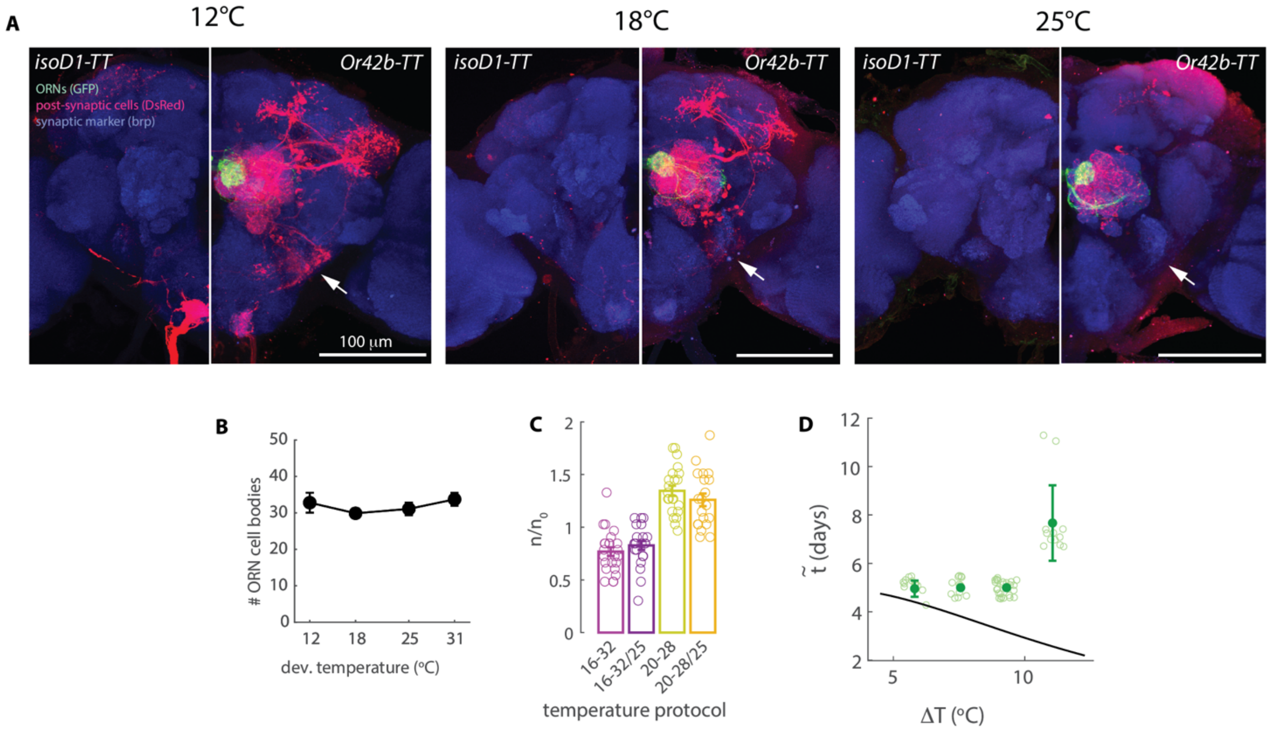

Fig. S2. Development at extreme and cycling temperatures. (A) Sample brains developed at three different temperatures show trans-Tango labeling of new cell types (arrows) connected to *Or42b*-ORNs at low temperatures. Images for 18 and 25°C are reproduced from Figure 1B. In flies developed at 12°C, trans-Tango labels neurons that innervate the saddle (SAD), one of the four SEZ subregions, which is not labeled at other temperatures. The left images are from genetic controls for the trans-Tango tool crossed to a control line (isoD1). **(B)** Mean number of ORNs cell bodies as a function of temperature (error bars indicate SEM, N=9-11). Note that this figures also includes data shown in main Fig. 4B. **(C)** Fold change in synaptic partners from 25°C for development on cycling temperatures. Temperature manipulations were applied only between L3 and P-100% then flies were moved to 25°C, except for the light-purple (16-32) and light-yellow (20-28) bars for which flies were kept through adulthood at the cycling temperatures (N=10-12). **(D)** Developmental times (adult eclosion) for pupae developed on temperature cycles of different amplitude (ΔT, mean temperature was 24°C) (N=12-25). The black line indicates the model prediction. Developmental times deviates from the model prediction, despite the nice agreement of the model with the measured connectivity (Fig. 2Q of main text), indicating a disruptive effect of temperatures above 30°C.

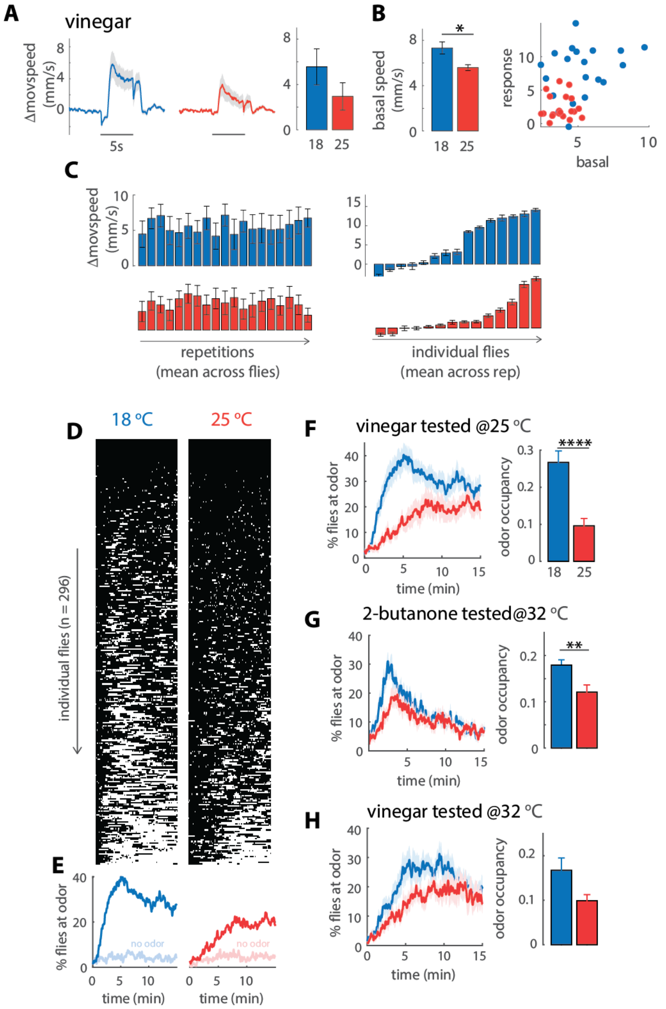

Fig. S3. Effects of developmental temperature on odor driven behavior. A-C) Response to vinegar on the spherical treadmill setup for flies developed at 18°C (blue) and 25°C (red). **(A)** Odor response calculated as change in moving speed as a function of time in response to 5s stimulation; bar plot: mean change in moving speed within the first 2s of stimulation ($p = 0.1$, N=14,13). **(B)** Left: mean basal walking speed is estimated from the 3s preceding stimulus onset ($p = 0.02$). Right: scatter plot of basal walking speed vs odor response for individual flies, showing that in both conditions basal and response behaviors are not correlated: both flies with high and low basal speed can respond strongly to the odor. **(C)** Mean response to each consecutive stimuli repetitions averaged across all flies (right), and mean response of each individual fly across repetitions, showing large variation across individuals (left). **(D-H)** Behavioral response in the free walking assay. **(D)** Binary maps showing individual flies located at the odor source (<5 cm, white) as a function of time and **(E)** percentage of flies at the odor (vinegar). An empty odor vial was used as control in an independent set of experiments (shaded curves). **(F)** Average percentage of flies at the odor source and odor occupancy (bar plot), for vinegar tested at 25°C ($p<10^{-4}$, N=21,20). **(G)** same as in (f) for a testing temperature of 32°C for 2-butanone ($p=0.006$, N=20,20) and **(H)** vinegar ($p=0.09$, N=19,18). Shaded area and error bars indicate SEM.

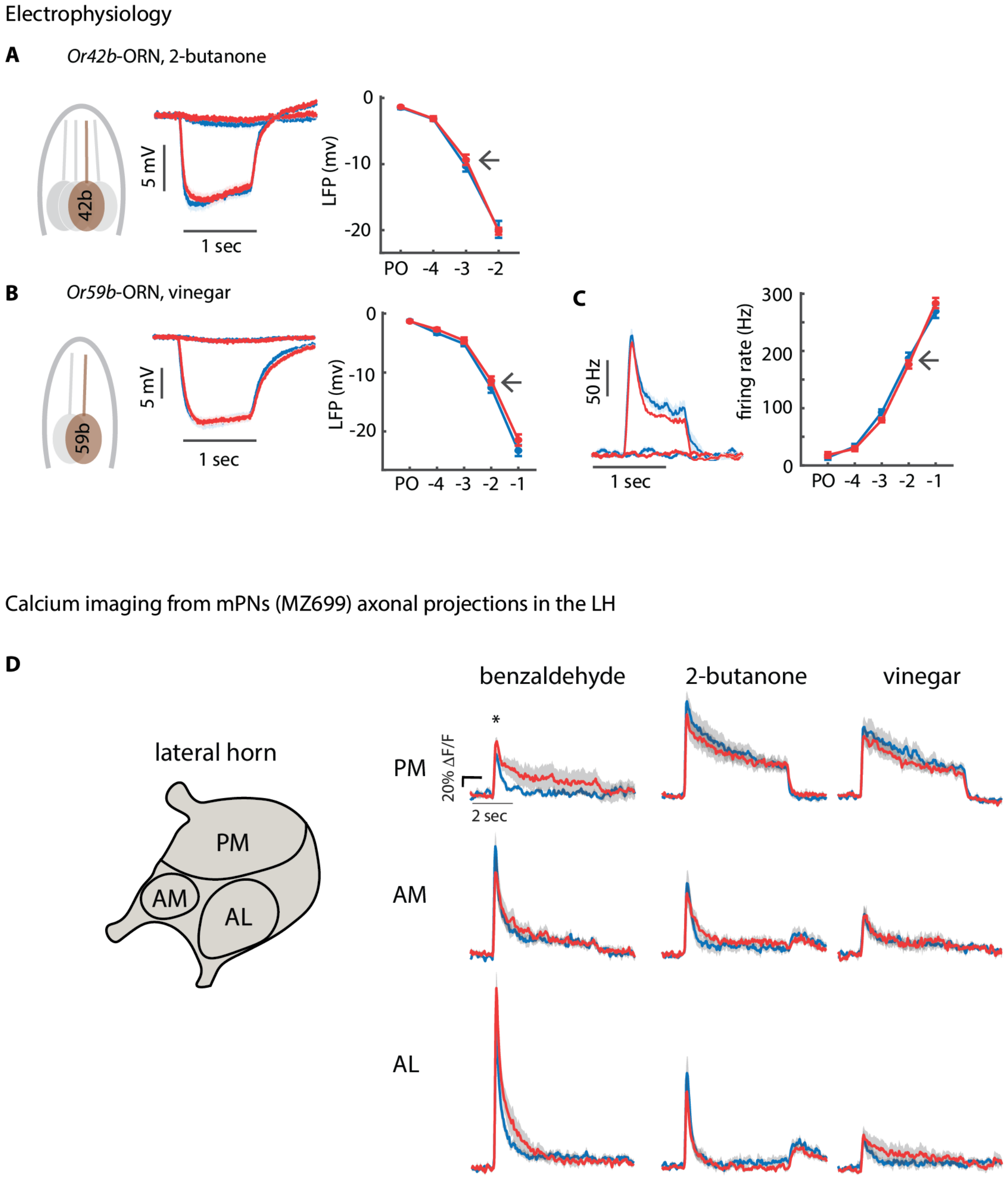

Fig. S4. Effects of developmental temperature on odor encoding. (A) Left to right: Single sensillum recordings from the ab1 sensillum containing a single *Or42b*-ORN. Mean response of the LFP to a 1s pulse of 2-butanone at 10^{-3} dilution and Paraffin Oil (PO) control. Amplitude of the LFP calculated as the maximum drop for each odor concentration. **(B)** Same as for the ab2 sensillum containing one *Or59b*-ORN. **(C)** Firing rate response of ab2 to a 1s pulse of 2-butanone at 10^{-3} dilution and PO control calculated in 100ms sliding window. Right: mean peak response for each concentration tested. **(D)** Calcium imaging from the axon terminals of mPNs labeled by *MZ699-Gal4*. For functional quantification, the LH was divided in regions based on Strutz et al. (24): posterior-medial (PM), anterior-medial (AM) and anterior-lateral (AL). As previously reported the posterior-medial LH is more sensitive to appetitive odors (N=6-10). All response curves are mean responses and shaded areas are SEM (*p<0.05).

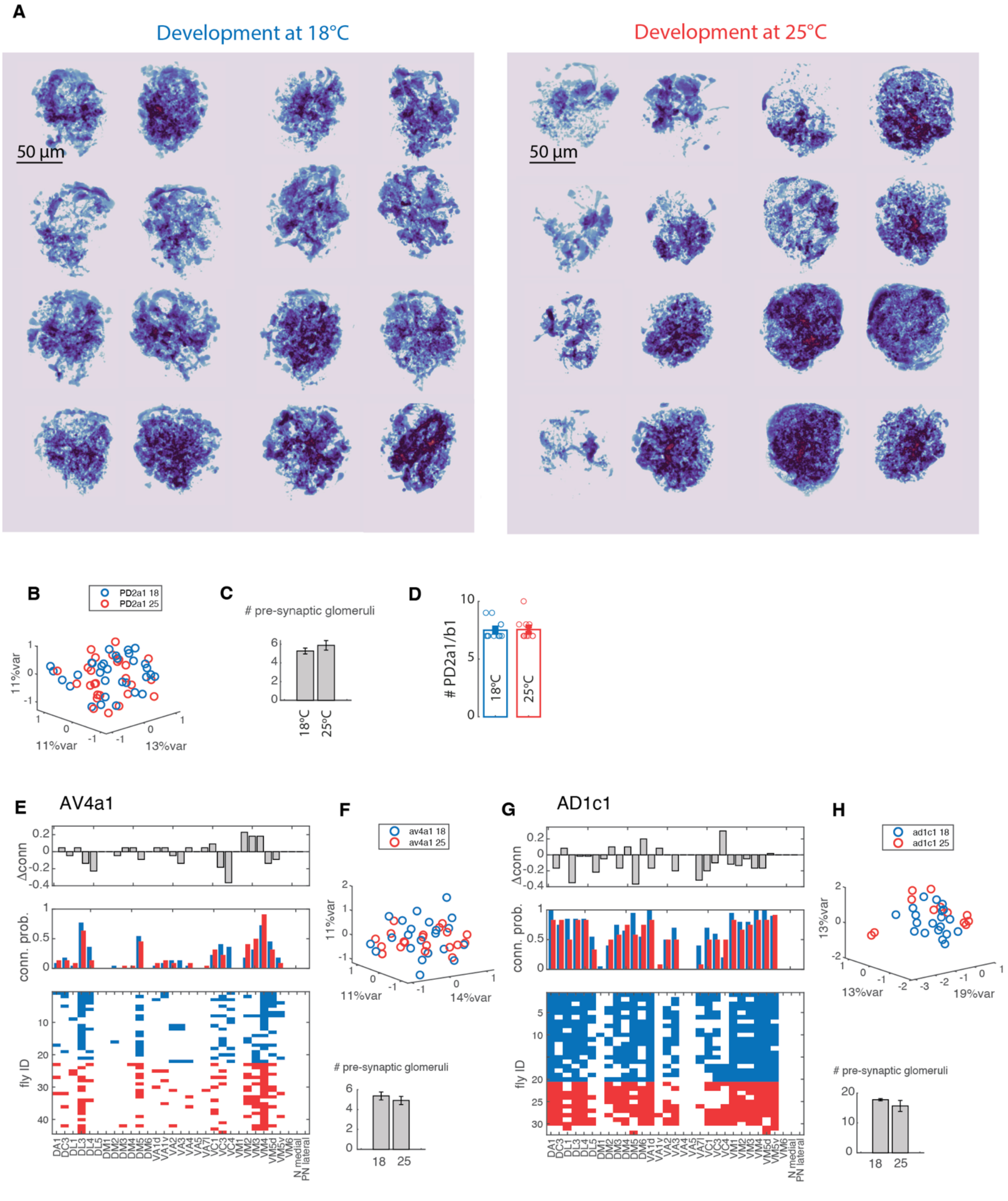

Fig. S5. Connectivity analysis of LH neurons. (A) Dendritic arborizations of neurons pre-synaptic to PD2a1/ as labeled by retro-Tango in individual brains. All brains developed at 18°C show stronger and more uniform innervation of the AL as compared to brain that developed at 25°C, which have more asymmetric innervations of the two hemibrains. **(B)** PCA of connectivity matrix of PD2a1/b1 to the AL glomeruli as quantified from BAcTrace in main Fig. 5G: each dot indicates a single hemibrain**.** (**C**) Mean number of pre-synaptic glomeruli for PD2a1/b1 in each hemibrain. (**D**) Bar plot showing mean number of PD2a1/b1 neurons labeled by the split-Gal4 line at the two developmental temperatures (error bars are SEM, N=12/11 hemibrains). (**E and G)** As main Fig.

5C-D-E for AV4a1 and AD1c1. Bottom: binary connectivity matrix. Middle: Connection probability to each glomerulus. Top: difference in connectivity between 25°C and 18°C. Middle and top panel are the same as in Fig. 5J. (**F and H**) As (C) and (D) for AV4a1 and AD1c1.

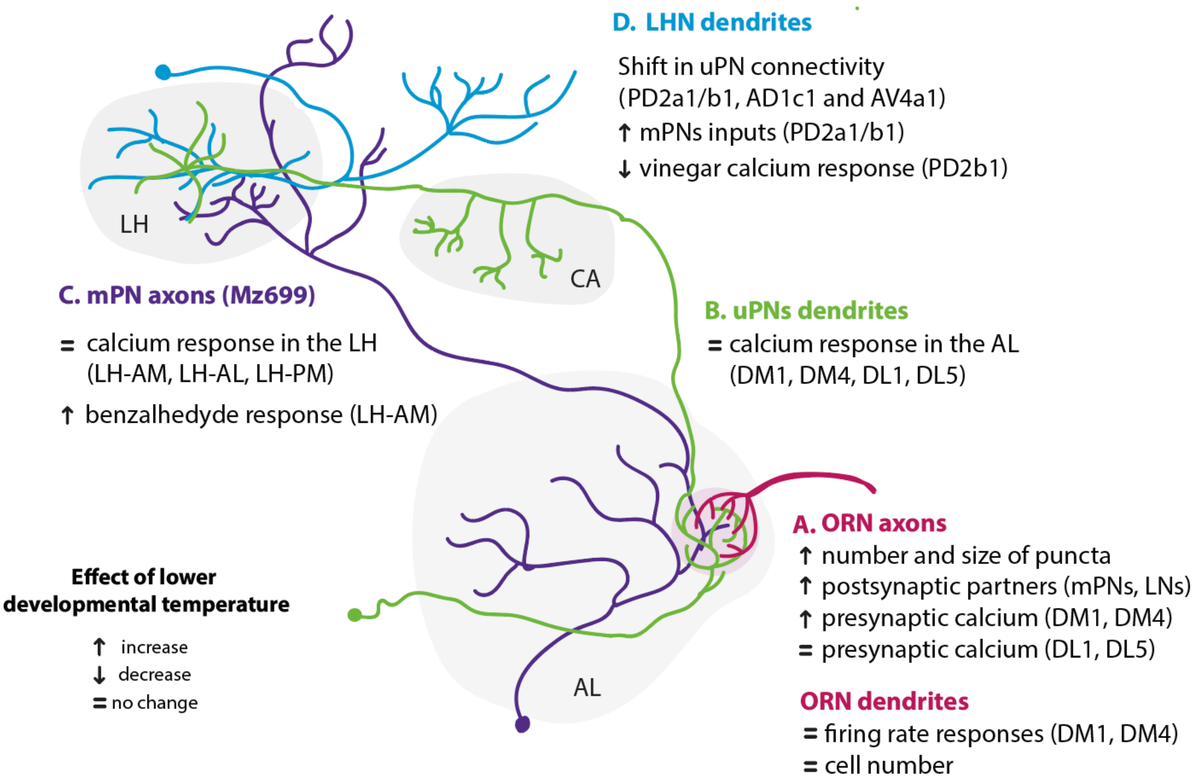

Fig. S6. Overview of study findings throughout the olfactory system. Changes are indicated as found in flies developed at 18°C relative to 25°C. Subpanels indicate different system levels analyzed. **(A)** ORN axons and dendrites, **(B)** uPNs dendrites, **(C)** mPNs axons, and **(D)** LHNs dendrites.

Reagent or Resource	Source	Identifier
**Antibodies**		
chicken anti-GFP	Abcam	Abcam Cat# ab13970, RRID:AB_300798
rabbit anti-DsRed	Takara Bio	Takara Bio Cat# 632496, RRID:AB_10013483
Mouse anti-nc82	deposited to the DSHB by Buchner, E.	DSHB Cat# nc82, RRID:AB_2314866
Goat anti-chicken Alexa Fluor 488	Jackson Immunoresearch	Jackson ImmunoResearch Labs Cat# 103-545-155, RRID:AB_2337390
Donkey anti-rabbit Alexa Fluor 568	Thermo Fisher Scientific	Thermo Fisher Scientific Cat# A10042, RRID:AB_2534017
Donkey anti-mouse Alexa Fluor 647	Thermo Fisher Scientific	Thermo Fisher Scientific Cat# A-31571, RRID:AB_162542
**Chemicals**		
PBS	GIBCO	Art. #70011044
Paraformaldehyde	Polysciences	Art. #00380-1
Triton X-100	Roth	Art. #3051.2
Normal goat serum	Thermo Scientific	Art. #31872
VectaShield	Biozol	Art. #VEC-H-1000
Apple Cider Vinegar	Ja! REWE, Apfel Essig	N/A
2-butanone	Sigma-Aldrich Co.	Art. #34861-M, CAS 78-93-3
Benzaldehyde	Sigma-Aldrich Co.	Art. #B1334, CAS 100-52-7
Mineral Oil	Sigma-Aldrich Co.	Art. #330779-1L, CAS 8042-47-5
All trans retinal	Sigma-Aldrich Co.	Art. #R2500
Sigmacote®	Sigma-Aldrich Co.	Art. #SL2
**Experimental Models: Organisms/Strains**		
D. melanogaster: *UAS-brpD3::GFP*	Gift from R. Heisinger	N/A
D. melanogaster: *Or42b-Gal4*	Bloomington Drosophila Stock Center (BDSC)	RRID: BDSC_9972
D. melanogaster: *Or7a-Gal4*	BDSC	RRID: BSDC_23907
D. melanogaster: Or10a-Gal4	BDSC	RRID: BSDC_9944
D. melanogaster: trans-Tango	BDSC	RRID: BSDC_77124

D. melanogaster: QUAS-GCamp6s; trans-Tango	BDSC	RRID: BSDC_95316
D. melanogaster: PD2a1/b1 split-Gal4 (R48F03-p65AD; R37G11-Gal4DBD)	BDSC	RRID: BSDC_86654
D. melanogaster: BAcTrace (w^+,QUAS-mtdTomato_UAS-CD2(attp8); pJFRC19-Syb::GFP-P10(VK37) VT033006-LexAp65(JK22C) LexAop-minQf-noV5-minSNAP25-HIVNES-Syntaxin(VK18);P{20XUASI-B3R.PEST}attp2 pJFRC161-B3RT-B2B3RT-HBMBoNTa2xGFPnb(VK5) QUAS-mtdTomato::HA)	Gift from S. Cachero	N/A
D. melanogaster: GH146-Gal4	Gift from A. Fiala	N/A
D. melanogaster: Orco-Gal4	BDSC	RRID: BSDC_23292
D. melanogaster: isoD1	Gift from M. Silies	N/A
D. melanogaster: UAS- GCaMP6f	BDSC	RRID: BSDC_42747
D. melanogaster: UAS-CsChrimson	BDSC	RRID: BSDC_82181
D. melanogaster: retro-Tango	BDSC	RRID: BSDC_99661
**Software and algorithms**		
ImageJ		RRID:SCR_003070
Leica Application Suite X	Leica	RRID:SCR_013673
MATLAB	MathWorks	RRID:SCR_001622
Adobe Illustrator	Adobe	RRID:SCR_010279
Hemibrainr (v. 0.1.0)	*(21)*	https://github.com/natverse/hemibrainr
Pylon viewer (64-Bit, 6.3.0.10295)		
FicTrac	*(64)*	
Trex 1.1.8_3	*(65)*	RRID:SCR_022361
Python 3.9.12		RRID:SCR_008394
RStudio		RRID:SCR_000432
Napari-pyclesperanto	*(60)*	https://github.com/clEsperanto/pyclesperanto_prototype

Supplementary Table 1. Resources table for all used antibodies, chemicals and experimental organisms used in this study, with their respective source and identifier.

Name	Genotype	Figure
Or42b > trans-Tango	*w+, UAS-myrGFP.QUAS-mtdTomato-3xHA; trans-Tango / CyO ; Or42b-Gal4 / +*	Fig. 1B-D, Fig. 1M, Fig. 2A-E, Fig. S1B,C, Fig S2
Or42b > trans-Tango QUAS-GCaMP6s	*w+ 10x QUAS-GCaMP6s; trans-Tango / UAS-CsChrimson; or42b-Gal4 / +*	Fig. 1F-G, Fig S.1A
Or42b > Brp^{Short} GFP	*;+;UAS-BRP D3::GFP/or42b-Gal4*	Fig. 1I-J, Fig S.1D
Or7a > trans-Tango	*w+, UAS-myrGFP.QUAS-mtdTomato-3xHA; trans-Tango / Or7a-Gal4*	Fig. 1N
Or10a > trans-Tango	*w+, UAS-myrGFP.QUAS-mtdTomato-3xHA; trans-Tango / Or10a-Gal4*	Fig. 1O
GH146 > GCaMP6f	*w+; UAS-GCaMP6f/ GH146-Gal4*	Fig. 2N,O<br>Fig. 4D
Or42b > GFP	*w+, UAS-myrGFP.QUAS-mtdTomato-3xHA; trans-Tango / CyO ; or42b-Gal4 / +*	Fig. 4B
Orco > GCaMP6f	*w+; UAS-GCamp6f / +; orco-Gal4 / +*	Fig. 4A,C, Fig. S4A-B
PD2a1/b1 > *BacTrace*	*w+, QUAS-mtdTomato,UAS-CD2/+; Syb::GFP, VT033006-LexAp65, LexAop-minQf-noV5-minSNAP25-HIVNES-Syntaxin/R48F03-p65AD; UASI-B3R.PEST,B3RT-B2B3RT-HBMBoNTa2xGFPnb, QUAS-mtdTomato::HA / R37G11-Gal4DBD*	Fig. 5E-I, Fig. S5B-D
PD2a1/b1 > *GCaMP6f*	*w+; UAS-GCaMP6f/ R48F03-p65AD ; R37G11-Gal4DBD / +*	Fig. 5K
AD1c1 > *BacTrace*	*w+, QUAS-mtdTomato,UAS-CD2/+; Syb::GFP, VT033006-LexAp65, LexAop-minQf-noV5-minSNAP25-HIVNES-Syntaxin/R55C09-GAL4; UASI-B3R.PEST,B3RT-B2B3RT-HBMBoNTa2xGFPnb, QUAS-mtdTomato::HA / +*	Fig. 5J, Fig. S5G,H
AV4a1 > *BacTrace*	*w+, QUAS-mtdTomato,UAS-CD2/+; Syb::GFP, VT033006-LexAp65, LexAop-minQf-noV5-minSNAP25-HIVNES-Syntaxin/ R34C08-p65.AD; UASI-B3R.PEST,B3RT-B2B3RT-HBMBoNTa2xGFPnb, QUAS-mtdTomato::HA / R22C12-GAL4DBD}*	Fig. 5J, Fig. S5E,F
PD2a1 > *retro-Tango*	*y w+ QUAS-mtdTomato-3xHA; retro-Tango / + ; 10xUAS-retro-Tango/ R48F03-p65AD; R37G11-Gal4DBD / +*	Fig. 5A-D, Fig. S5A
isoD1 > *trans-Tango*	*w+, UAS-myrGFP.QUAS-mtdTomato-3xHA/isoD1; trans-Tango / isoD1*	Fig. S2A
MZ699 > GCaMP8f	*w+; UAS-GCaMP8f/+; MZ699-Gal4/+*	Fig. S4D

Supplementary Table 2. Fly strains used in the paper, separated by used name, full genotype and figure in which it appears.
